# Neurodegenerative Diseases – Is Metabolic Deficiency the Root Cause?

**DOI:** 10.3389/fnins.2020.00213

**Published:** 2020-03-31

**Authors:** Vignayanandam Ravindernath Muddapu, S. Akila Parvathy Dharshini, V. Srinivasa Chakravarthy, M. Michael Gromiha

**Affiliations:** ^1^Laboratory for Computational Neuroscience, Department of Biotechnology, Bhupat and Jyoti Mehta School of Biosciences, Indian Institute of Technology Madras, Chennai, India; ^2^Protein Bioinformatics Lab, Department of Biotechnology, Bhupat and Jyoti Mehta School of Biosciences, Indian Institute of Technology Madras, Chennai, India

**Keywords:** metabolic deficiency, selective vulnerability, excitotoxicity, mitochondrial dysfunction, oxidative stress, protein mishandling, glia-vascular integrity, insulin resistance

## Abstract

Neurodegenerative diseases, including Alzheimer, Parkinson, Huntington, and amyotrophic lateral sclerosis, are a prominent class of neurological diseases currently without a cure. They are characterized by an inexorable loss of a specific type of neurons. The selective vulnerability of specific neuronal clusters (typically a subcortical cluster) in the early stages, followed by the spread of the disease to higher cortical areas, is a typical pattern of disease progression. Neurodegenerative diseases share a range of molecular and cellular pathologies, including protein aggregation, mitochondrial dysfunction, glutamate toxicity, calcium load, proteolytic stress, oxidative stress, neuroinflammation, and aging, which contribute to neuronal death. Efforts to treat these diseases are often limited by the fact that they tend to address any one of the above pathological changes while ignoring others. Lack of clarity regarding a possible root cause that underlies all the above pathologies poses a significant challenge. In search of an integrative theory for neurodegenerative pathology, we hypothesize that metabolic deficiency in certain vulnerable neuronal clusters is the common underlying thread that links many dimensions of the disease. The current review aims to present an outline of such an integrative theory. We present a new perspective of neurodegenerative diseases as metabolic disorders at molecular, cellular, and systems levels. This helps to understand a common underlying mechanism of the many facets of the disease and may lead to more promising disease-modifying therapeutic interventions. Here, we briefly discuss the selective metabolic vulnerability of specific neuronal clusters and also the involvement of glia and vascular dysfunctions. Any failure in satisfaction of the metabolic demand by the neurons triggers a chain of events that precipitate various manifestations of neurodegenerative pathology.

## Introduction

Neurodegenerative diseases, including Alzheimer (AD), Parkinson (PD), Huntington (HD), and Lou Gehrig or amyotrophic lateral sclerosis (ALS), are characterized by inexorable degeneration of specific neural clusters. This class of diseases poses a serious clinical challenge because once the neurodegeneration begins, it can only be slowed down but not fully halted (Fu et al., 2018). Typically, the progression of neurodegeneration starts in the subcortical regions and spreads to cortical regions as the disease progresses (Zhou et al., 2012). The primary neuronal loss varies with the disease such as striatal regions in PD, striatal and cortical regions in HD, hippocampal and cortical regions in AD, and spinal motor neurons and cortical regions in ALS (Ilieva et al., 2009; Marambaud et al., 2009; Kiaei, 2013).

Neurodegenerative diseases share a range of molecular and cellular pathologies, including protein aggregation, mitochondrial dysfunction, glutamate toxicity, calcium load, proteolytic stress, oxidative stress, neuroinflammation, and aging, resulting in neuronal death (Kiaei, 2013; Gan et al., 2018). In these diseases, there are specific neuronal clusters that are primarily vulnerable, which become the original site from which the pathology spreads (Fu et al., 2018). These vulnerable neurons have complex morphological features such as long-range neuronal projections and extensive synaptic connections, which makes them selectively vulnerable due to their higher metabolic demands to maintain their structural complexity (Bolam and Pissadaki, 2012; Pissadaki and Bolam, 2013; Pacelli et al., 2015).

We hypothesize that energy deficiency occurs at different neural hierarchies, such as subcellular, cellular, and systems levels (Bolam and Pissadaki, 2012; Pissadaki and Bolam, 2013; Pacelli et al., 2015; Muddapu et al., 2019), may be a root cause of neuronal loss. We propose that the pathological markers of neurodegenerative diseases, such as mitochondrial dysfunctions, protein mishandling, and oxidative stress, are a direct consequence of metabolic abnormalities (Song and Kim, 2016). Contrarily, energy deficiency can also be triggered by oxidative stress, glutamate toxicity, and excessive calcium load. We also looked into whether protein aggregation is a cause or a symptom of energy deficiency. There may exist a bidirectional relationship between genetic dysfunctions and metabolic abnormalities. However, most cases of neurodegenerative disorders seem to be sporadic, and usually, a definite genetic cause might not be established (Shin et al., 2011; Brahmachari et al., 2019; Zambon et al., 2019). The known pathologies of neurodegenerative diseases are listed in Table 1. Viewing these disorders as a result of metabolic abnormalities can open up new avenues of therapeutic research to protect the neuronal population and improve outcomes for patients.

**TABLE 1:** Summarizing the known pathologies in neurodegenerative diseases (Ilieva et al., 2009; Marambaud et al., 2009; Federico et al., 2012; Johri and Beal, 2012; Ravisankar et al., 2018; Sweeney et al., 2018a; Castelli et al., 2019).

Pathology	PD	AD	HD	ALS
Protein aggregation	α-Synuclein	β-Amyloid, tau	Huntingtin (htt)	Superoxide dismutase 1 (SOD1), FUS, TDP-43, OPTN, UBQLN2
Mitochondrial complexes dysfunction	I, IV, V	I, II, III, IV, V	I, II, III, IV, V	I, II, III, IV, V
Proteins affecting mitochondrial function	Tau, α-synuclein, parkin, PINK1, DJ-1, LRRK2, HTRA2	Amyloid precursor protein, presenilin (PS1, PS2), β-amyloid, tau	htt	SOD1
Factors causing calcium homeostasis dysregulations	NMDAR (slow inactivation), α-synuclein pores, mitochondrial abnormalities, underexpression of calcium-buffering proteins	NMDAR (slow inactivation), β-amyloid pores, mitochondrial abnormalities, underexpression of calcium-buffering proteins	NMDAR (slow inactivation), mitochondrial abnormalities, underexpression of calcium-buffering proteins, over sensitization of InsP3R	AMPAR (no GluR2 subunit), mitochondrial abnormalities, underexpression of calcium-buffering proteins
Contributors to oxidative stress	α-Synuclein, mitochondrial dysfunction, impaired neurotrophins	β-amyloid, tau, mitochondrial dysfunction, impaired neurotrophins	α-Synuclein, htt, mitochondrial dysfunction	SOD1, mitochondrial dysfunction
Glial impairment	Astrocytes, microglia, oligodendrocytes	Astrocytes, microglia	Astrocytes, microglia	Astrocytes, microglia, oligodendrocytes
Vascular dysfunctions	CBF reduction, impaired cerebrovascular reactivity, increased BBB permeability, microbleeds, diminished P-glycoprotein function	CBF reduction, impaired cerebrovascular reactivity, impaired neurovascular coupling, increased BBB permeability, microbleeds, diminished glucose transport, and metabolism, diminished P-glycoprotein function	CBF reduction, increased BBB permeability	CBF reduction, microbleeds

Current clinical treatments aim at managing clinical symptoms rather than curing the underlying pathological cause (Chen and Pan, 2015). Several US Food and Drug Administration (FDA)–approved treatments available for different neurodegenerative diseases are listed in Table 2. However, these drugs exhibit side effects, such as gait disturbance, rigidity, tremor, hives, headache, drowsiness, nausea, and so on (Durães et al., 2018). This shows that, although current medications can reduce the symptoms, there are no permanent solutions to rescue the surviving neuronal population. The leading cause of the selective vulnerability is still unclear. To determine potential drug targets and develop novel therapeutic strategy, it is essential to identify the underlying cause of the disease. In this review, we briefly discuss the selective metabolic vulnerability of specific neuronal clusters and the role of metabolic stress related to various pathological events. Because of structural complexity, the vulnerable neurons have higher energy demand, which they try to meet through oxidative phosphorylation. The increased production of adenosine triphosphate (ATP) and depletion of antioxidants induce reactive oxygen species (ROS) response that leads to oxidative stress, which in turn influences the function of mitochondria. Furthermore, aberrant glutamate signaling leads to excessive calcium load that additionally induces metabolic and endoplasmic reticulum (ER) stress. Any slight perturbation in the energy metabolism affects these neurons tremendously. From this literature background, we propose a plausible mechanism for selective neurodegeneration, which argues that an imbalance between energy supply and demand impacts these specific neurons at a higher rate compared to other neuronal populations.

**TABLE 2:** FDA-approved drugs for different neurodegenerative diseases.

Pathology	Act as	Effective	Drug(s)
PD	Dopamine supplement	Most potent in the case of bradykinesia	Levodopa
	3, 4-dihydroxyphenylalanine (DOPA) decarboxylase inhibitor	Improves therapeutic benefits of levodopa	Carbidopa, benserazide
	Catechol-o-methyltransferase (COMT) inhibitor	Improves therapeutic benefits of levodopa	Tolcapone, entacapone
	Monoamine oxidase (MAO) inhibitor	Improves therapeutic benefits of levodopa	Selegiline, rasagiline
	Dopamine agonist	Delayed onset of dyskinesia	Apomorphine (rescue drug), pramipexole
	Antagonist of NMDA receptor	Antidyskinetic drug	Amantadine
	Anticholinergics drug	Young patients dominated by tremor	Trihexyphenidyl, benztropine
	Adenosine antagonist	Antidyskinetic drug	Istradefyline
AD	Acetyl cholinesterase inhibitor	Dementia	Donepezil, rivastigmine, galantamine
	Antagonist of NMDA receptor		Memantine
ALS	Antagonist of NMDA receptor	Slow down disease progression	Riluzole
	Antioxidant		Edaravone
HD	Antichorea drug	Chorea	Tetrabenazine

## Selective Metabolic Vulnerability of Specific Neuronal Populations

**FIGURE 1:**
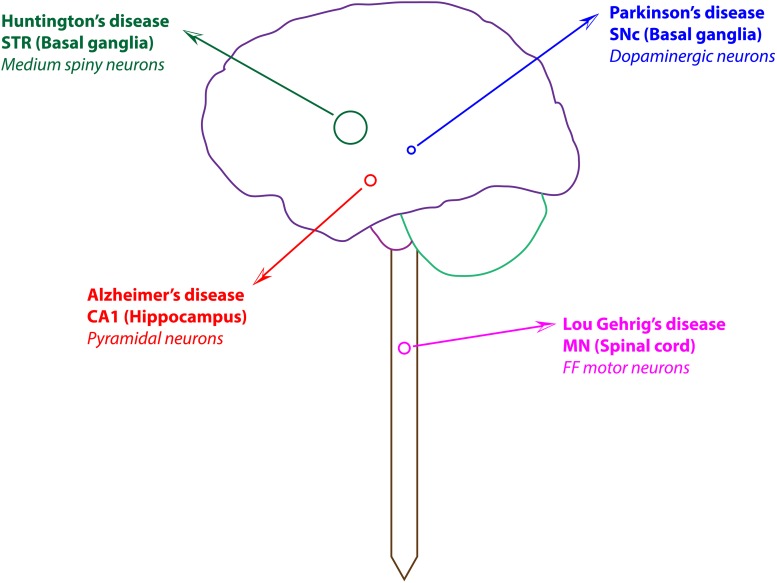
Selective vulnerable neuronal population in various neurodegenerative disorders. STR, striatum; SNc, substantia nigra pars compacta; CA1, cornu ammonis 1; MN, motor neuron; FF, fast fatigable.

In various neurodegenerative disorders, a specific population of neurons is selectively vulnerable because of their structural and functional complexity (Figure 1). In case of PD, there is a significant loss of dopaminergic neurons, specifically in the substantia nigra pars compacta (SNc) region (Figure 1). However, dopaminergic neurons, which are abundantly present in other regions, such as ventral tegmental area (VTA) and olfactory bulb, are affected to much lesser extent (Hirsch et al., 1988; German et al., 1989, 1992; Dauer and Przedborski, 2003; Pacelli et al., 2015). What is the distinctive vulnerability of SNc neurons compared to neurons in the VTA? The possible reason could be that SNc neurons possess complex arborization and extensive synaptic connections (Bolam and Pissadaki, 2012; Brichta and Greengard, 2014), which result in a higher basal metabolic rate and increased oxidative stress compared to other closely related dopaminergic neurons such as those of VTA and olfactory bulb (Pissadaki and Bolam, 2013; Pacelli et al., 2015).

In AD, hippocampal neurons located in the cornu ammonis 1 (CA1) field are selectively vulnerable (Wilde et al., 1997; Padurariu et al., 2012a; Figure 1). In AD patients, the volume of the neuronal population in the CA1 region is significantly smaller than in other regions (CA2, CA3) of the hippocampus (Padurariu et al., 2012a). Electrophysiology studies showed that the overall firing rate of the CA1 population is higher than that of the CA3 population, which makes CA1 neurons a more energy-craving neuronal population compared to other hippocampal fields (Mizuseki et al., 2012). Thus, under ischemic stress, a higher number of neurons in the CA1 region were susceptible when compared to the CA3 region in the rat brain (Wilde et al., 1997; De Jong et al., 1999). Anatomical studies showed differences between the vascular architecture in CA1 and CA3 regions; CA1 region contains a large ventral artery, whereas a higher number of capillaries were found in the vicinity of CA3 neurons (Duvernoy et al., 2013). These vascular features can be linked to the higher energy requirements of CA1 neurons compared to CA3 neurons.

In ALS, Fast-Fatigable (FF) motor neurons, which are a subset of α motor neurons, are specifically vulnerable compared to other motor neurons (Figure 1; Conradi and Ronnevi, 1993; Pun et al., 2006; Kaplan et al., 2014). As this subgroup of motor neurons involved in intense movements such as jumping and running, they require an enormous amount of energy for their functioning, which makes them vulnerable under metabolic abnormalities (Brandstater and Lambert, 1973; Hegedus et al., 2007). Moreover, because of their enormous axonal arborization and large axonal diameter when compared to other closely related slow motor neurons, FF motor neurons require a greater amount of energy to maintain and propagate information across their complex axonal structures (Kawamura et al., 1981; Kernell and Zwaagstra, 1981; Hegedus et al., 2007; Stifani, 2014). Electrophysiology studies show that the overall firing rate of FF motor neurons was higher when compared to slow motor neurons because of shorter after-hyperpolarization latency following an action potential, which makes FF motor neurons a more energy-craving neuronal population compared to others (Gardiner, 1993). As a result of higher energy requirements of FF motor neurons, they are prone to higher ER stress compared to slow motor neurons (Saxena et al., 2009). Thus, it indicates that FF motor neurons tend to be more vulnerable when compared to slower motor neurons in similar stress conditions.

In HD, enkephalin-positive medium spiny neurons (MSNs) of striatum are selectively vulnerable (Reiner et al., 1988; Albin et al., 1992; Richfield et al., 1995; Figure 1). The MSNs (GABAergic/enkephalin) projecting to globus pallidus externa (GPe) through the indirect pathway tend to die earlier in disease pathogenesis (Reiner et al., 1988; Albin et al., 1992; Richfield et al., 1995). The striatal MSNs and cortical pyramidal neurons (projection neurons) are more vulnerable to degeneration due to their peculiar long axonal arborization that projects to distant regions in the brain (Cudkowicz and Kowall, 1990; Hedreen et al., 1991; Her and Goldstein, 2008). Contrarily, the striatal and cortical interneurons are less vulnerable due to their extensive dendritic network that projects locally when compared to projection neurons, which have long-range projections (Morigaki and Goto, 2017). To maintain this complex axonal arborization, projection neurons require enormous energy, much more than a typical neuron. In HD, enkephalin levels are reduced (Augood et al., 1996; Poulin et al., 2014) during pathogenesis, but overexpressing pre-enkephalin alleviates HD symptoms (Bissonnette et al., 2013). The polyglutamine-huntingtin (polyQ-Htt)–induced toxicity does not occur selectively in any specific neuronal population, but cell-specific features might differentially render specific neuronal populations increasingly vulnerable to polyQ-Htt–induced toxicity (Han et al., 2010). However, it is still not clear why enkephalin-positive MSNs are more vulnerable in HD compared to substance P positive MSNs.

Several studies reported that mitochondrial-derived energy failure inhibits synaptic vesicle recycling in presynaptic boutons, which leads to neurodegeneration in hippocampus and striatum (Pathak et al., 2015; Kayser et al., 2016; Adami et al., 2017; Devine and Kittler, 2018; Reeve et al., 2018; Restelli et al., 2018). The accumulation of α-synuclein in mitochondria was reported in substantia nigra and striatum in PD patients, which can hinder mitochondrial functions (Devi et al., 2008). It has been reported that the cerebral metabolic rate of glucose was reduced by ∼25% in case of AD patients (Kalaria and Harik, 1989; Hock et al., 1997). It was reported that glucose consumption was reduced in the brain, especially in the basal ganglia in HD patients and presymptomatic mutation carriers also (Grafton et al., 1992; Kuwert et al., 1993; Antonini et al., 1996). The energy homeostasis in ALS patients was observed to be disrupted (Dupuis et al., 2011; Vandoorne et al., 2018), where energy uptake was decreased, and energy consumption was increased (Bouteloup et al., 2009; Ahmed et al., 2016), which results in reduced fat deposits in ALS patients (Huisman et al., 2015). Thus, these studies support the idea of energy deficiency as a cause of neurodegeneration.

In summary, all the aforementioned neurodegenerative diseases indicate that vulnerable neurons have high energy demands due to their anatomical complexity and rich synaptic connectivity. These regions act as energy-craving hubs, and deterioration in the energy supply could lead to neurodegeneration.

## Is the Selective Vulnerability in the Neuronal Population Related to Oxidative Stress?

The neurons susceptible to degeneration need higher energy to maintain their structural and functional integrity. Therefore, any disruption in energy metabolism can lead to enormous energy demand that eventually results in cellular stress. Because mitochondria are vital for oxidative energy metabolism, mitochondrial dysfunction is a major contributor to metabolic stress. Increased production of ATP results in higher ROS production. Inadequate antioxidants, scavenging enzymes, and functional abnormalities in the mitochondria can cause oxidative stress (Su et al., 2010). This oxidative stress affects mitochondrial DNA (mtDNA) and leads to an aberration in mitochondrial quality control and electron transport chain enzymes, which results in metabolic deficiency. Furthermore, this may also result in broken cristae and other structural abnormalities in mitochondria. Along with morphological abnormalities in mitochondria, increased calcium influx and overproduction of ROS may accelerate the formation of inner mitochondrial transition pore (MTP), which leads to apoptosis (Liu et al., 1996; Halestrap et al., 1997; Castilho et al., 1998; Pacelli et al., 2015).

### Parkinson Disease

SNc dopamine (DA) neurons exhibit elevated rates of oxidative phosphorylation, showing a threefold increase in ATP production and ROS generation in SNc compared to VTA neurons (Pacelli et al., 2015). A higher density of mitochondria in SNc axonal arbor compared to VTA indicates a higher energy requirement for the functioning of SNc neurons (Pacelli et al., 2015; Giguère et al., 2019).

### Alzheimer Disease

Higher levels of superoxide and ROS were observed in the CA1 region compared to the CA3 region from *in vitro* studies (Wilde et al., 1997; Wang et al., 2005). This result shows that there is an aberrant oxidative phosphorylation mechanism in CA1 neurons, which results in augmented ROS production. *In vivo* studies demonstrate that ROS and calcium-induced stress in the CA1 region activates MTPs at higher rate, leading to calcium-induced mitochondrial swelling compared to the CA3 region (Mattiasson et al., 2003). These studies illustrate that these two neuronal populations respond differently to similar stress conditions. In rodent studies, severe mitochondrial damages were observed in the CA1 region when compared to the CA3 region in post-ischemic conditions (Radenovic et al., 2011). Thus, neurons in the CA1 region are more susceptible to mitochondrial damage and oxidative stress when compared to the CA3 region (Kanak et al., 2013). Human gene expression studies reveal that genes involved in energy metabolism were downregulated in AD. Also, reduced activity of glycolytic and oxidative phosphorylation enzymes was observed in AD patient samples (Iwangoff et al., 1980; Perry et al., 1980; Sorbi et al., 1983; Kish et al., 1992; Akila Parvathy Dharshini et al., 2019). Thus, these studies suggest that CA1 neurons are more susceptible to oxidative stress because of their enormous energy requirements.

### Amyotrophic Lateral Sclerosis

In order to study the role of mitochondria in ALS pathogenesis, mitochondrial enzymatic activity was recorded from various neuronal tissues (brain and spinal cord) of mice expressing human superoxide dismutase 1 (SOD1) mutant (hSOD1-G93A). They found that cytochrome oxidase (complex IV) and ROS-scavenging activity were significantly lower in motor neurons, which resulted in increased mtDNA deletions (Mattiazzi et al., 2002). A large number of mtDNA deletions were observed in ALS patients, which might be due to reduced ROS-scavenging activity (Swerdlow et al., 1998; Vielhaber, 2000; Menzies et al., 2002). The reduced axonal mitochondria were associated with aberrant axonal transport (De Vos et al., 2007) as a result of aggregated toxic SOD1 interfering with the functioning of dynein motor proteins (Zhang et al., 2007), which are involved in retrograde transport (i.e., it transports the damaged mitochondria or misfolded protein from the axonal terminal to the soma for degradation). Besides, SOD1 mutant mice showed that increased ROS response impairs energy production as well as inhibits the cargo activity as a consequence of higher energy demand. This might be due to increased level of ROS, which in turn impairs both mitochondrial functionality and protein degradation, thereby inhibiting the cargo activity.

### Huntington Disease

Functional assay studies have demonstrated that rat striatal mitochondria are more selective in forming MTPs in response to ROS and calcium-induced stress (Brustovetsky et al., 2003) than the mitochondria in the cortical neurons. Furthermore, HD knock-in studies illustrate that ROS and reactive nitrogen species (RNS) were predominately observed in striatal cells and impact the electron transport chain (Ribeiro et al., 2013). A greater mitochondrial mass was found to be present in the striatum, and significant mtDNA deletions were observed in HD transgenic mice when compared to other brain regions as a result of reduced ROS-scavenging activity (Hering et al., 2015). The gene expression studies of HD patients showed increased levels of antioxidants and severe defects in the activity of the respiratory chain complexes (II, III, and IV) in striatum as a consequence of oxidative stress (Gu et al., 1996; Sorolla et al., 2008).

In summary, the characteristic features among all the above neurodegenerative diseases include increased number of mitochondria in vulnerable neuronal populations, increased ROS responses, aberrant respiratory chain enzymes, and higher ATP production. In general, these neurons require more substantial amount of energy to perform the electrical-spiking activity, even small perturbations in energy production mechanisms and mitochondrial functioning significantly affect these neuronal populations compared to others. Calcium-induced stress and increased ROS response impair mitochondrial functionality by disrupting mtDNA, which in turn affects mitochondrial morphology and oxidative energy metabolism. All these events form MTPs, thereby leading to apoptosis. Transgenic mice studies showed that overexpression of peroxisome proliferator activator receptor and γ-coactivator 1α exhibits increased mitochondrial biogenesis, which improves neuronal activity and electron transport energy metabolism (Zhao et al., 2011). Restoration of mitochondrial function may thus improve neuronal survivability by reducing cellular stress.

## How Do Glutamate Toxicity and Calcium Load Affect Mitochondrial Function?

**FIGURE 2:**
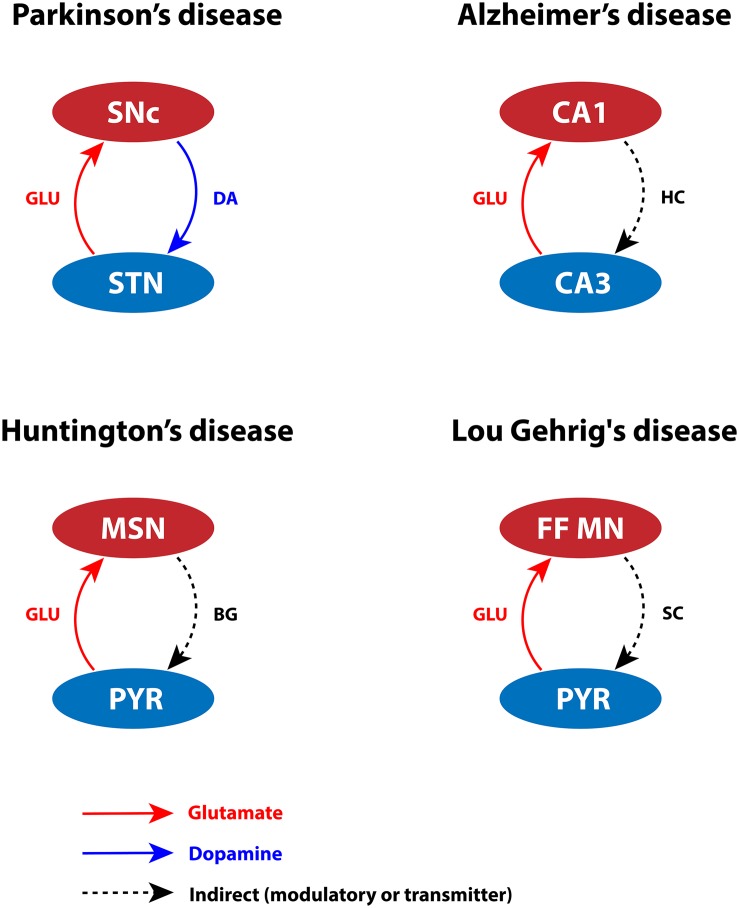
Glutamate-induced excitotoxicity in various neurodegenerative disorders. SNc, substantia nigra pars compacta; STN, subthalamic nucleus; DA, dopamine; GLU, glutamate; CA1, cornu ammonis 1; CA3, cornu ammonis 3; HC, hippocampus; MSN, medium spiny neuron; PYR, pyramidal neuron; BG, basal ganglia; FF MN, fast fatigable motor neuron; SC, spinal cord.

Glutamate is an essential excitatory neurotransmitter that can lead to toxicity when present at high concentrations. Glutamate toxicity may manifest at different levels in the brain (such as subcellular, cellular, or systems level). At subcellular level, differential expression of calcium-binding (CBP) and calcium-sensing proteins can lead to inadequate calcium-buffering capacity, resulting in oversensitivity of neurons to glutamate released extracellularly (German et al., 1992; Gaspar et al., 1994; Liang et al., 1996; Damier et al., 1999; Foehring et al., 2009). At cellular level, the glutamate concentration is affected by differential expression of glutamatergic and neuropeptide receptors. Additionally, extensive arboreal structures can tend to hyperexcitation of glutamate receptors even at the physiological levels of glutamate concentration (Pahapill and Lozano, 2000; Heng et al., 2009; Pacelli et al., 2015; Estakhr et al., 2017; Wang and Reddy, 2017; Giguère et al., 2019; Gregory et al., 2019). Finally, at systems level, overexcitation from pathogenic nuclei can lead to calcium accumulation in ER and mitochondria, which in turn results in neurodegeneration (Rodriguez et al., 1998; Hansson et al., 1999; Pahapill and Lozano, 2000; Kawahara et al., 2003; Vaarmann et al., 2013; Muddapu et al., 2019). Considering the factors mentioned above, we infer that certain types of cells might be more vulnerable in pathological conditions, as depicted in Figure 2.

### Parkinson Disease

An excess amount of glutamate damages SNc neurons by activating *N*-methyl-D-aspartate (NMDA) receptors (Kure et al., 1991; Tan et al., 2002; Mattson and Magnus, 2006). During the pace-making activity of SNc neurons, magnesium blockage of NMDA receptors becomes ineffective; as a result, a slight increase in glutamate stimulation creates a calcium storm in these neurons (Deister et al., 2009; Zweifel et al., 2009). This direct mechanism of toxicity is possible in case of acute neurological disorders such as ischemic/hypoxic damage to the brain (Rothman and Olney, 1986), but not in slowly evolving chronic disorders such as PD (Blandini, 2001). However, under energy deficit conditions, even physiological levels of glutamate are toxic as a result of increased intracellular calcium concentration. This leads to oxidative stress through a mechanism known as “indirect excitotoxicity or weak excitotoxicity” (Novelli et al., 1988; Albin and Greenamyre, 1992; Blandini, 2001). It was reported that glutamatergic excitation of SNc neurons by subthalamic nucleus (STN) neurons (Smith and Grace, 1992; Smith et al., 1996) under the conditions of bioenergetic deficiency may lead to aggravation of degeneration processes (Rodriguez et al., 1998; Connolly et al., 2014; Muddapu et al., 2019; Figure 2). The excitotoxic loss of SNc neurons might be due to disinhibition of STN (low DA) precipitated by energy deficiency (Muddapu et al., 2019). Apart from regulation by plasma membrane calcium pumps, ER, and mitochondria, intracellular calcium is also regulated by CBPs (calbindin and calmodulin), which are widely expressed in many brain regions (Liu and Graybiel, 1992). The presence of calbindin in midbrain DA neurons suggests being a marker to distinguish DA neurons with higher susceptibility to neurodegeneration in PD (Brichta and Greengard, 2014). The gene expression of calbindin was higher in VTA neurons when compared to SNc neurons in rats (Liang et al., 1996; Chung et al., 2005; Greene et al., 2005; Björklund and Dunnett, 2007) and humans (Mendez et al., 2005). Hence, lower levels of calbindin (German et al., 1992; Liang et al., 1996), heterogeneous expression of NMDA receptors, and overexcitation by STN may result in lower intrinsic calcium-buffering capacity (Foehring et al., 2009) of SNc neurons. Thus, inadequate calcium-buffering capacity may contribute to their selective vulnerability (Haddad and Nakamura, 2015).

### Alzheimer Disease

In case of CA1 neurons, the gene expression studies of AD-like rats showed differential expression of NMDA receptor subunit (NR1, NR2B) in the CA1 and CA3 regions (Liu et al., 2012). NR2B subunit is highly selective for calcium ion transport and is known to play a decisive role in calcium-induced apoptosis. In AD-like rats, it has been shown that NR2B was overexpressed in the CA1 subfield compared to controls (Liu et al., 2012). The persistent overactivation of NMDA receptor in the postsynaptic terminal stimulates CA1 neurons, which allows higher calcium influx resulting in excitotoxicity. During prolonged glutamate stimulation, calcium influx was higher in CA1 compared to CA3, and a substantial amount of calcium is sequestered in CA1 mitochondria (Stanika et al., 2010; Oh et al., 2013). Therefore, dysregulation of calcium homeostasis in the CA1 region forms MTPs, which disrupt the mitochondrial structural integrity and, in turn, affect mitochondrial functioning. Furthermore, it has been reported that CA3 neurons innervate glutamatergic projections to CA1 neurons known as Schaffer collaterals (Petersen et al., 1998). In AD, CA3 neurons are observed to be overactive, which may cause excitotoxic damage to CA1 neurons (Haberman et al., 2017; Figure 2). In aging rats, the reduction of calbindin expression was more significant in CA1 neurons compared to CA3 neurons (Potier et al., 1994; De Jong et al., 1996; Lee et al., 2013). In AD, the gene expression of neurofilament proteins and CBPs (parvalbumin, calbindin, and calretinin) was lower in CA1 neurons compared to CA3 neurons (Hof et al., 1996). Hence, lower levels of CBPs, heterogeneous expression of NMDA receptor subunits, and overexcitation by CA3 may result in lower intrinsic calcium-buffering capacity. Because of their inadequate calcium-buffering capacity, CA1 neurons are more prone to excitotoxic degeneration in AD.

### Amyotrophic Lateral Sclerosis

Excess glutamate in the synapse or extracellular space is transported into neuronal and glial cells via glutamate transporter excitatory amino acid carrier 1 and glutamate transporter 1 (GLT-1), respectively (Medina et al., 1996b; Förstl and Kurz, 1999). In healthy monkeys, it has been noted that increased expression of GLT-1 astroglial elements was observed in vulnerable FF motor neurons when compared to slow motor neurons. However, in case of ALS, lower expression of GLT-1 astroglial elements results in excess glutamate in the synaptic cleft, which leads to excitotoxicity in FF motor neurons (Medina et al., 1996b). In general, FF motor neurons are hyperexcitable (Gardiner, 1993; Wainger et al., 2014), and they receive glutamatergic projections from the pyramidal neurons of motor cortex (Nijssen et al., 2017). Moreover, in case of FF motor neurons, the ratio of excitatory to inhibitory synapses is higher, which makes them more vulnerable to excitotoxicity under metabolic abnormalities due to disrupted calcium homeostasis (Nijssen et al., 2017; Figure 2). The normal expression of CBPs, such as calmodulin, calbindin, and parvalbumin, is necessary to maintain calcium homeostasis (Alexianu et al., 1994). The expression of CBPs in FF motor neurons was lower when compared to slow motor neurons (Medina et al., 1996b), which may result in aberrant calcium regulation in FF motor neurons. The abundant glutamate activates α-amino-3-hydroxy-5-methyl-4-isoxazole propionic acid (AMPA) receptors, and increased presence of AMPA receptors was observed in spinal motor neurons (Van Den Bosch et al., 2000). Consequently, AMPA receptors allow greater calcium influx into the mitochondria. To regulate calcium levels, mitochondria require a large energy threshold that eventually leads to metabolic stress. It also produces ROS, which is toxic to spinal motor neurons (Carriedo et al., 2000). The studies mentioned above show that aberrant glutamate reuptake, dysfunction of calcium-buffering proteins, and increased glutamatergic drive cause overexcitation of postsynaptic neurons, which allow greater calcium influx, leading to disruption of neuronal metabolic functions.

### Huntington Disease

Striatal MSNs receive rich glutamatergic and dopaminergic inputs from cortex and SNc, respectively (Ehrlich, 2012). Any imbalance in neurotransmission might make MSNs more susceptible to glutamate-induced toxicity (Figure 2). In the early stages of HD, there is a loss of D2-type DA receptor (D_2_) binding in the striatum and increased GABA_*A*_ receptor binding in GPe, which suggests that medium spiny GABAergic/enkephalin neurons (D_2_ MSNs) in the indirect pathway are overexcited, leading to excitotoxicity (Glass et al., 2000). Any energy imbalance would increase stress on MSN, which can be exacerbated by polyQ-Htt, leading to (weak) excitotoxicity even at physiological concentrations of glutamate (Albin and Greenamyre, 1992; Estrada Sánchez et al., 2008). The expression of CBPs (calretinin, parvalbumin, and calbindin) is higher in striatal interneurons compared to the MSNs; these proteins may act as neuroprotective agents against calcium insults (Gerfen et al., 1985). In HD, the expression of calcium-sensing proteins, such as hippocalcin, reduced in MSNs compared to striatal interneurons, thereby increasing the vulnerability of MSNs (Luthi-Carter et al., 2000). The presence of calcium-binding and calcium-sensing proteins makes striatal interneurons less vulnerable than MSNs. The alterations in glutamate receptor trafficking and functionality, glutamate transport, lower intrinsic calcium-buffering capacity, and energy disruptions might together favor toward excitotoxic death of MSNs.

The vulnerability of specific neuronal populations in neurodegenerative diseases can be an accumulated effect of many factors across several levels of hierarchy from subcellular to systems level. There can be a positive feedback relationship between metabolic deficiency and disruption in some other factor (such as cell death), each reinforcing the other. A key player across multiple levels of hierarchy is calcium, which is essential for many cellular processes in the neuron. However, excess accumulation of calcium can lead to mitochondrial dysfunctions, oxidative stress, and protein mishandling (Post et al., 2018). In summary, the differential expression of glutamate transporters, glutamate receptors, and calcium-buffering proteins results in aberrant glutamate uptake, which leads to the sequential development of pathology as follows: overexcitation of the vulnerable neuronal population, imbalance in the calcium homeostasis, formation of MTP, mitochondrial swelling, and finally apoptosis.

## Is Protein Aggregation a Results or the Cause of Oxidative Stress?

One common element in all neurodegenerative diseases is the misfolded (or) damaged protein, which gets accumulated in the intracellular region of a neuron as an insoluble protein aggregate. The various stress factors influence protein aggregation, and aggregated proteins affect neuronal functionality, which results in cell death. Proteins undergo aggregation due to oxidative stress, excitotoxicity, and inflammation (Ross and Poirier, 2004; Gundersen, 2010). Predominantly, these aggregates are degraded by ubiquitin and lysosomal-mediated degradation processes. Increased oxidative stress influences the proteostasis and deteriorates ubiquitin-mediated proteasomal degradation mechanism. These protein degradation processes required higher levels of ATP for their normal functioning. The increased ROS and reduced ATP generation make the intracellular environment hostile enough to form protein aggregates. These aggregates affect vesicle transport and fuse with the mitochondrial membrane, which in turn increases the permeability that leads to mitochondrial swelling and apoptosis (Camilleri et al., 2013; Korovila et al., 2017).

In PD, healthy α-synuclein proteins may get misfolded because of mitochondrial dysfunction or oxidative stress or both, which in turn leads to formation of aggregates and Lewy bodies (LBs) (Gundersen, 2010). In the recent years, it has been claimed that a viral (Olsen et al., 2018), neurotoxic (Goldman, 2014) or prion-like infection (Olanow and Brundin, 2013; Matheoud et al., 2019) could be a cause of PD pathogenesis (Hawkes et al., 2007). There exist evidences supporting the Braak hypothesis of LBs (Braak et al., 2004), which suggests that the pathology (misfolded α-synuclein protein) spreads from peripheral regions of the nervous system to the central nervous system through transsynaptic transmission in PD (Braak et al., 2004; Chauhan and Jeans, 2015; Brundin et al., 2016; Brundin and Melki, 2017; Borghammer, 2018). The presence of LBs in grafted embryonic DA neurons in PD patients (Kordower et al., 2008; Li et al., 2008; Mendez et al., 2008) also supported this view of causation. To regard PD as a prion-like disease, it should satisfy one of the two rules – nearest neighbor rule or synaptic connectivity rule – but there is no clear evidence for the same, as discrete distribution patterns of LB pathology undermine the nearest neighbor rule within PD patients, and synaptic connectivity rule was not supported by connectomic data (Surmeier et al., 2010, 2017). Thus, there is no clear evidence to consider PD as a prion-like disease. However, α-synuclein aggregation might contribute to oxidative stress by inhibiting mitochondrial function (Di Maio et al., 2016). It was reported that mitochondrial-induced oxidative stress causes dysregulation of calcium and DA, which leads to α-synuclein aggregation and lysosomal dysfunction (Burbulla et al., 2017). Thus, oxidative stress might be caused by α-synuclein aggregation due to metabolic deficiency–derived dysfunctions of ROS-scavenging process. Contrarily, α-synuclein aggregation might be caused by oxidative stress due to metabolic deficiency.

In AD, β-amyloid and tau proteins are the major contributors to protein aggregation. β-Amyloid is involved in synaptic vesicle transport, and tau protein is essential for microtubule structural organization (Mietelska-Porowska et al., 2014). Due to environmental stress, these proteins tend to disrupt their structure and functionality, which results in formation of protein aggregates. β-Amyloid plaques form aggregates around neurons and mitochondria, which disrupt their functions (Su et al., 2010). These plaques tend to trigger dysfunction of various mitochondrial proteins that, in turn, affect the cell morphology. Further, additional energy is required to disintegrate the aggregation. The age-dependent glucose hypometabolism (de Leon et al., 1983) and oxidative stress lead to ATP decline in neurons (Hof and Morrison, 2004). Therefore, metabolic stress caused by energy demand leads to dysregulation of lysosomal, proteasomal-mediated degradation mechanism, resulting in higher propensity of protein aggregation in the neuron (Gabuzda et al., 1994; Gasparini et al., 1997).

It has been reported that familial mutant SOD1 forms a toxic aggregate, which impairs Na-K-ATPase α3 activity by interacting with its nucleotide-binding site, thereby interfering with neural firing activity (Ruegsegger et al., 2016). Furthermore, Na^+^/K^+^ Na-K-ATPase α3 is abundant in spinal motor neurons. Thus, misfolded protein tends to lose its structural features and form an insoluble aggregate, which induces cellular stress and eventually disrupts neuronal function. The defective protein is usually tagged with ubiquitin for proteasomal degradation, which requires ATP. Ubiquitin C-terminal hydrolase L (UCHL1) is essential for the cerebrospinal muscular junction. It was reported that reduced activity of UCHL1 is associated with defective ubiquitination process, which, in turn, results in increased ER stress (Genç et al., 2016). Furthermore, lowered neuronal energy metabolism results in dysregulation of ATP-mediated protein degradation.

In HD, genetic instability of Htt gene translates into mutant protein Htt, which contains disease-causing expansions of glutamines that make them misfold and form aggregates (Arrasate and Finkbeiner, 2012). These aggregates bind to mitochondria resulting in oxidative stress. In striatum, MSNs are less enriched by superoxide free radical scavengers such as SOD1 and SOD2 when compared to cholinergic interneurons (Medina et al., 1996a). This difference probably makes MSNs more prone to oxidative stress, which contributes to protein aggregation (Medina et al., 1996a).

In summary, protein aggregation is a common phenomenon in normal cells. These proteins are broken down by various degradation processes. Under energy deficiency conditions, neurons possess high energy requirements that lead to excess ROS build-up and exacerbate inflammatory response. Depletion of ATP and protein degradation mechanisms may induce the propensity of protein aggregation, which is deleterious to neuronal function.

## The Role of Glial Cells in Neuroinflammation and Glutamate-Induced Toxicity

Microglia are also referred to as the military of the brain because they are highly mobile, can sense a threat, and fight against it (Nayak et al., 2014). The increased ROS and tissue injury activate resting microglia by sensing the signals released by damaged neurons. Microglia play dual roles – they can be a friend or a foe to brain tissue. Under normal conditions, they nurture and sustain the neural tissue by providing neurotrophic, neuroprotective, and neurotoxic factors. Under pathological conditions, the overactivated microglia produce higher amount of inflammatory cytokines that can cause neuronal death (Miller et al., 2013). Microglia also activate reactive astrocytes by forming glial scars, which protect healthy tissue from inflammatory signals. Growing experimental evidence demonstrates that reactive gliosis blocks regeneration of axons, synaptic formation, and phagocytic activity by releasing neurotoxic substances (Liddelow et al., 2017).

From animal models of PD, it was observed that the pattern of cell loss was regulated by the level of oxidative stress caused by inflammation (Teismann et al., 2003; Hunot et al., 2004; Koprich et al., 2008). In human subjects, it was suspected that inflammation and microglial activation might contribute to disease progression in late stages (Tansey and Goldberg, 2010). Astrocytes play a modulatory role in microglial activation (McGeer and McGeer, 2008; Glass et al., 2010; Rocha et al., 2012). Miscommunication between astrocytes and microglia results in neuroinflammation, which eventually leads to neurodegeneration (Waak et al., 2009; Booth et al., 2017). It has been reported that neuromelanin can induce microglial activation (Zecca et al., 2008; Zhang et al., 2011). Substantia nigra pars compacta neurons are more susceptible to neuromelanin-induced inflammation compared to VTA neurons because of their high neuromelanin biosynthesis (Peter et al., 1995; Liang et al., 2004). It has also been reported that cyclooxygenase-2 (*COX-2*) gene is overexpressed in SNc neurons, which are classic proinflammatory mediators, and leads to degeneration in PD (Dauer and Przedborski, 2003; Teismann et al., 2003). From these pieces of evidence, it may be understood that SNc neurons are prone to be more vulnerable to PD pathogenesis compared to other brain regions.

In AD, reactive gliosis occurs at higher levels in the CA1 region compared to others (Rodríguez et al., 2013; Kamphuis et al., 2014). Due to metabolic stress, CA1 astrocytes exhibit increased ROS response, decreased mitochondrial fidelity, and reduced activity of glutamate transporters, leading to tremendous stress in CA1 neurons (Ouyang et al., 2007). Several researchers hypothesized that because of metabolic stress, astroglia turns into inflammatory cells, relinquishing their neurosupportive roles (Fuller et al., 2010). Activated glial cells are also potential sources of ROS and RNS. Overproduction of nitridergic species, such as peroxynitrite and nitric oxide, is said to be dependent on activated glial cells in addition to the mitochondria. Astrocytes interact with endothelial cells and maintain the blood–brain barrier (BBB) permeability (Alvarez et al., 2013; Sweeney et al., 2018b). During pathological conditions, astrocytes release cytokines that disrupt BBB permeability (Abbott, 2002). All these events in the brain lead to disturbed homeostasis and drive disease progression.

Microglia, astrocytes, oligodendrocytes, and Schwann cells play vital roles in disease progression as glial pathology is observed in all cases of familial and sporadic ALS (Kushner et al., 1991; Keller et al., 2009). Microgliosis is found to be an early and typical hallmark of the disease. In ALS, astrogliosis leads to dysfunction in the glutamate transporter system that results in excitotoxic motor neuron cell death. Post-mortem studies of ALS patients revealed reduced numbers of oligodendrocytes, which leads to reduced capacity to release lactate, causing oxidative stress (Lee et al., 2012; Philips et al., 2013; Philips and Rothstein, 2014). Such defects lead to glutamate excitotoxicity, glia cell pathology, mitochondrial damage, and defective axonal transport. Because astrocytes are devoid of their calcium-signaling mechanisms in ALS, elevated calcium levels tend to disrupt the mitochondria and induce oxidative stress, which leads to higher tendency of apoptosis mediated by cytochrome c.

Astrocytes play a vital role in HD when compared to microglia. Studies showed that striatal astrocytes were unable to handle the calcium and glutamate signaling in HD (Jiang et al., 2016). An increased amount of glutamate in the synaptic cleft may induce glutamate-mediated excitotoxicity. Calcium mishandling leads to an increased calcium influx inside the cell and disrupts the mitochondrial morphology. Mutant Htt in astrocytes and neurons elevate the microglial response, which induces cytotoxicity (Shin et al., 2005; Khakh, 2019).

Hence, glia–neuron interactions can intensify pathogenesis in the brain and interfere with signaling pathways and normal metabolism. Gliodegenerative events in neurodegenerative diseases disrupt the calcium homeostasis and glutamate transport and induce an inflammatory response. These events cause havoc in the brain, which correlates to disease conditions. In summary, mitochondrial bioenergetics and glial pathology can be interlinked in the pathogenesis of neurotoxicity.

## How Does Vascular Dysfunction Affect the Energy Metabolism?

Because the cerebrovascular network is the source of energy substrates of neural tissue, metabolic deficiency in the neural tissue can be traced to disruptions in cerebrovascular function. The BBB, which serves as a gateway between the cerebrovascular systems and the brain, comprises tight junctions of endothelial cells, pericytes, and basal membrane. Blood–brain barrier is essential to preserve the brain against pathogenic invaders. The vascular region supplies the metabolites and neurotrophic factors to energy-demanding neurons directly via the medium of extracellular space and indirectly via the glial cells (Philips and Rothstein, 2014). Studies on various neurological disorders report that impairment of BBB and the vascular network lead to neuroinflammation, increased endothelial cell permeability, reduced glucose transport, and invasion of toxic substances, which eventually affect neurovascular communication and cerebral blood flow (CBF) (Abbott, 2002; Zhu et al., 2007; Miyazaki et al., 2011; Freeman and Keller, 2012; Guan et al., 2013; Sweeney et al., 2018a, b). Cerebral blood flow is an essential source of blood glucose supply and helps to preserve functional homeostasis of the neuron. Studies have shown reduced regional CBF in AD (Dai et al., 2009), PD (Derejko et al., 2001), HD (Hasselbalch et al., 1992), and ALS (Kew et al., 1993).

In a classic experiment aimed to study the role of oxidative stress in various hippocampus subfields (Chip et al., 2013), oxygen supply to various neuronal subfields was reduced and then measured the blood vessel count in normal and stress-subjected neuronal populations. Because of ischemic stress, the number of blood vessels drastically reduced in CA1 compared to other regions. This demonstrates that there is a regional vascular vulnerability associated with the CA1 region, which in turn depletes the blood supply to the specific subregion. In ALS, studies have shown discrepancies in barrier conditions, especially in the pericyte region (Garbuzova-Davis and Sanberg, 2014), which leads to increased pinocytosis and affects BBB integrity. From the above studies, it was observed that BBB integrity was disturbed in various neurodegenerative disorders. Blood–brain barrier disruption leads to impairment in vascular–glial communication, resulting in energy crisis (Alvarez et al., 2013), and impacts the functioning of energy-craving neurons. Because of increased energy demand, these vulnerable neurons are unable to meet their energy requirements for securing functional homeostasis.

## How Does Insulin Resistance Affect Vascular Integrity and Energy Metabolism?

Insulin is a primary regulating hormone in the human body, which plays a crucial role in glucose and lipid metabolisms. Lower levels of insulin are observed in cerebrospinal fluid in contrast to plasma in AD (Craft et al., 1998). Abnormalities in insulin receptors and their sensitivity are observed in PD (Marques et al., 2018), ALS (Perurena and Festoff, 1987), and HD (Lalić et al., 2008). However, this insulin resistance leads to an increased amount of advanced glycation end products and upregulates neuroinflammatory signals (nuclear factor κB, tumor necrosis factor α, cytokines), causing vascular injury as well as dysregulate nitric oxide production in endothelial cells, which impairs the vasodilatory effects (Goldin et al., 2006). Age-dependent insulin resistance dysregulates the insulin receptor abundance and glucose transporters in BBB, which depletes glucose supply to the neuron, in turn, leading to glucose hypometabolism (Craft, 2007, 2009; Willette et al., 2015). Insulin regulates cholesterol metabolism that is essential for myelination and regulation of amyloid protein degradation enzymes (Wang et al., 2014). Impairment of insulin regulation upregulates low-density lipoproteins and downregulates high-density lipoproteins, which results in amyloid aggregation (Reitz, 2013). Insulin resistance results in an imbalance in glucose metabolism (disruption of endothelial barrier integrity) by causing abnormalities in glucose uptake and oxidation, decreasing the synthesis of glycogen, and reducing the ability to suppress lipid metabolism. Aberrant glucose homeostasis causes chronic hyperglycemia, which results in oxidative stress (aberrant mitochondrial function and nitric oxide synthase (NOS) phosphorylation). Increased oxidative stress results in an inflammatory response by activating microglia and reactive astrocytes, which in turn result in cellular damage (Bhatti et al., 2017; Lee et al., 2018; Ormazabal et al., 2018).

## Role of Aging

Aging plays a vital role in determining the progression of neurodegenerative disorders (Padurariu et al., 2012b). During aging, SNc (Fearnley and Lees, 1991), CA1 (Kusindarta et al., 2018), and striatal (Umegaki et al., 2008) neuronal populations tend to diminish. In some cases, studies have shown selective degeneration of synaptic inputs in FF motor neurons, as opposed to cell death (Maxwell et al., 2018). To understand regional vulnerability, researchers measured protein kinase B (also known as AKT) gene expression level. AKT is essential for neuronal survival, growth, and angiogenesis, which is downregulated in AD (Jackson et al., 2009; O’ Neill, 2013), PD (Timmons et al., 2009), and HD (Humbert et al., 2002). However, reduced amount of AKT shows a significant increase in proapoptotic signals (FOX3a). Furthermore, changes in AKT expression may be a potential pathway to induce apoptosis (Jackson et al., 2009). Age-related glucose hypometabolism, morphological structures, and insulin resistance affect these neurons drastically and lead to an energy crisis.

During typical aging, it has been reported that SNc neurons exhibit compensatory changes (hypertrophy), which result in normal motor function despite cell loss. However, in case of PD, this compensatory mechanism is compromised, resulting in impaired motor function (Rudow et al., 2008). The vulnerability of SNc DA neurons in PD is further amplified by reduced expression of calbindin in aging (Mattson and Magnus, 2006). There is no clear justification for why aging is a significant risk factor in PD. However, there are several speculations that deteriorating mitochondrial function might be a significant factor that results in hypometabolism (Schapira, 2008; Büeler, 2009; Van Laar and Berman, 2009; Boumezbeur et al., 2010; Winklhofer and Haass, 2010).

During aging, the CA3 region is hyperactivated, whereas the CA1 region showed a reduced firing rate (Kanak et al., 2013; Simkin et al., 2015; Oh et al., 2016). As we age, overexcitation of excitatory neurons leads to an excessive accumulation of glutamate in the synaptic cleft (Esposito et al., 2013; Assefa et al., 2018), which results in glutamate-induced toxicity. In aging, larger numbers of reactive astrocytes (A1) are observed in CA1 and striatal regions that, in turn, induce neuroinflammation and vulnerability in pyramidal neurons and MSNs of CA1 and striatal regions, respectively (Clarke et al., 2018). Studies have shown age-dependent increase in the β-amyloid, α-synuclein, SOD1, and Htt protein aggregation (Kennedy, 2000; Ishiguro et al., 2001; Lee et al., 2011; Tomé and Dandelot, 2017; Medinas et al., 2018, 2019). Because of the energy crisis, this type of aggregation leads to enormous stress in the specific neuronal classes.

## Proposed Hypothesis for Selective Neurodegeneration

**FIGURE 3:**
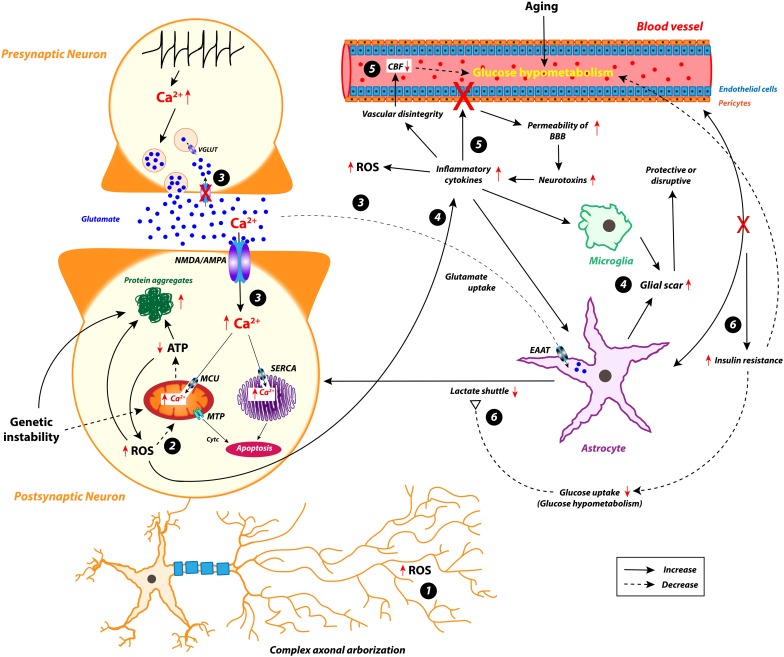
The plausible hypothesis for selective neurodegeneration in various neurodegenerative disorders. EAAR, excitatory amino acid receptors; MCU, mitochondrial calcium uniporter; SERCA, sarcoplasmic/endoplasmic reticulum Ca^2+^-ATPase; EAAT, excitatory amino acid transporters; CBF, cerebral blood flow; Ca^2+^, calcium; ATP, adenosine triphosphate; ROS, reactive oxygen species. BBB, blood-brain barrier; Cytc, cytochrome c; MTP, mitochondrial transition pore; NMDA, N-methyl-D-aspartate; AMPA, α-amino-3-hydroxy-5-methyl-4-isoxazolepropionic acid; VGLUT, vesicular glutamate transporter.

From the literature review presented in this article, we propose a causative hypothesis for selective neurodegeneration (Figure 3).

1. Typically, in any neurodegenerative disease, the surviving selectively vulnerable neuronal population retains complex axonal arbors and large number of synaptic connections that exhibit elevated energy demand for maintaining the structural and functional integrity.
2. Neurons mainly depend on oxidative phosphorylation for their elevated energy requirements. Because of increased energy demand, mitochondria tend to produce excess ROS. The depletion of antioxidants alleviates ROS level above a certain threshold, which subsequently affects the mitochondrial morphology by forming MTP and leads to metabolic stress.
3. Impaired glutamate transporter function in the glial cell leads to excessive glutamate accumulation in the synaptic cleft that activates postsynaptic AMPA/NMDA receptors. This allows increased calcium influx in postsynaptic neurons, disrupting calcium homeostasis in the mitochondria and ER and producing excessive levels of ROS.
4. Because of metabolic stress, the degenerative neurons release inflammatory cytokines, which can be sensed by astrocytes and microglia. However, the reactive astroglia forms a glial scar, which can protect as well as attack the stressed/injured neuronal population. The increased inflammatory response can also lead to excess ROS formation.
5. The increased inflammatory and ROS responses may strain the endothelial cells and pericytes in BBB and result in increased permeability of the membrane, which in turn allows toxic invaders inside the brain. These events further enhance inflammatory response, disrupt vascular integrity, and reduce regional CBF in various neurodegenerative diseases (Dharshini et al., 2019).
6. The communication loss between glia and vascular systems leads to insulin resistance that additionally induces inflammation and reduces glucose uptake, which leads to glucose hypometabolism. Moreover, the reduced levels of glucose influence the lactate shuttle, which in turn results in diminished oxidative phosphorylation.

All the above events impose significant metabolic stress on the neural populations that are already selectively vulnerable due to their distinct structural and functional characteristics. Along with this energy crisis, additional cytotoxic factors, such as protein aggregation, genetic risk factors, and calcium load, expand the vulnerability of these neurons at a higher rate compared to others. This shows an imbalance between energy supply and demand, which seriously challenges the survivability of the energy-craving neuron and leads to selective neurodegeneration.

## Conclusion

Neurons that are selectively vulnerable to metabolic deficiency possess complex arboreal structures and functional responsibilities and therefore require an enormous amount of energy for their survival. The increased production of ATP leads to an increase in ROS formation in mitochondria, which indirectly affects the mitochondrial quality control and respiratory chain enzymes (energy metabolizing enzymes), and, in turn, leads to metabolic stress. Furthermore, dysfunction in glutamate receptors and transporters may hyperexcite postsynaptic neurons, which in turn causes calcium accumulation inside the mitochondria and ER that drives neuronal cells to metabolic stress. In addition, damaged/injured neurons send inflammatory signals that lead to activation of microglia and reactive astrocytes. The increased immune response may affect endothelial cells in BBB that impacts vascular integrity and cerebral blood pressure, which in turn leads to reduced CBF and increased insulin resistance. This may contribute toward glucose hypometabolism, resulting in lesser lactate uptake and affecting oxidative phosphorylation in the vulnerable neuron. Along with this tremendous metabolic stress, an additional toxic substance, protein aggregation, and glutamate excitotoxicity may affect these energy-craving neurons at a higher rate compared to other neurons. Based on the above observations, we propose an integrative theory for neurodegeneration, which suggests that these neuronal populations crave for energy, and any deterioration in the energy supply may perturb these neurons immensely. Restoration of metabolic function may save these vulnerable neurons from going down the path of neurodegeneration.

## Author Contributions

VM and SD equally contributed to writing the main text and gathering all references. VC and MG contributed to editing the manuscript drafts, providing insight into structure, and material that should be included.

## Conflict of Interest

The authors declare that the research was conducted in the absence of any commercial or financial relationships that could be construed as a potential conflict of interest.

## References

[B1] Abbott N. J. (2002). Astrocyte-endothelial interactions and blood-brain barrier permeability. *J. Anat.* 200 629–638. 10.1046/j.1469-7580.2002.00064.x 12162730 PMC1570746

[B2] Adami P. V. M. Quijano C. Magnani N. Galeano P. Evelson P. Cassina A. (2017). Synaptosomal bioenergetic defects are associated with cognitive impairment in a transgenic rat model of early Alzheimer’s disease. *J. Cereb. Blood Flow Metab.* 37 69–84. 10.1177/0271678X15615132 26661224 PMC5363729

[B3] Ahmed R. M. Irish M. Piguet O. Halliday G. M. Ittner L. M. Farooqi S. (2016). Amyotrophic lateral sclerosis and frontotemporal dementia: distinct and overlapping changes in eating behaviour and metabolism. *Lancet Neurol.* 15 332–342. 10.1016/S1474-4422(15)00380-4 26822748

[B4] Akila Parvathy, Dharshini S. Taguchi Y. Michael Gromiha M. (2019). Exploring the selective vulnerability in Alzheimer disease using tissue specific variant analysis. *Genomics* 111 936–949. 10.1016/j.ygeno.2018.05.024 29879491

[B5] Albin R. L. Greenamyre J. T. (1992). Alternative excitotoxic hypotheses. *Neurology* 42 733–738. 10.1212/WNL.42.4.733 1314341

[B6] Albin R. L. Reiner A. Anderson K. D. Dure L. S. Handelin B. Balfour R. (1992). Preferential loss of striato−external pallidal projection neurons in presymptomatic Huntington’s disease. *Ann. Neurol.* 31 425–430. 10.1002/ana.410310412 1375014

[B7] Alexianu M. E. Ho B. −K. Mohamed A. H. La Bella V. Smith R. G. Appel S. H. (1994). The role of calcium−binding proteins in selective motoneuron vulnerability in amyotrophic lateral sclerosis. *Ann. Neurol.* 36 846–858. 10.1002/ana.410360608 7998770

[B8] Alvarez J. I. Katayama T. Prat A. (2013). Glial influence on the blood brain barrier. *Glia* 61 1939–1958. 10.1002/glia.22575 24123158 PMC4068281

[B9] Antonini A. Leenders K. L. Spiegel R. Meier D. Vontobel P. Weigell-Weber M. (1996). Striatal glucose metabolism and dopamine D2 receptor binding in asymptomatic gene carriers and patients with Huntington’s disease. *Brain* 119 2085–2095. 10.1093/brain/119.6.2085 9010012

[B10] Arrasate M. Finkbeiner S. (2012). Protein aggregates in Huntington’s disease. *Exp. Neurol.* 238 1–11. 10.1016/j.expneurol.2011.12.013 22200539 PMC3909772

[B11] Assefa B. T. Gebre A. K. Altaye B. M. (2018). Reactive astrocytes as drug target in Alzheimer’s Disease. *Biomed Res. Int.* 2018:4160247. 10.1155/2018/4160247 29888263 PMC5977027

[B12] Augood S. J. Faull R. L. M. Love D. R. Emson P. C. (1996). Reduction in enkephalin and substance P messenger RNA in the striatum of early grade Huntington’s disease: a detailed cellular in situ hybridization study. *Neuroscience* 72 1023–1036. 10.1016/0306-4522(95)00595-1 8735227

[B13] Bhatti J. S. Bhatti G. K. Reddy P. H. (2017). Mitochondrial dysfunction and oxidative stress in metabolic disorders — A step towards mitochondria based therapeutic strategies. *Biochim. Biophys. Acta Mol. Basis Dis.* 1863 1066–1077. 10.1016/j.bbadis.2016.11.010 27836629 PMC5423868

[B14] Bissonnette S. Vaillancourt M. Hébert S. S. Drolet G. Samadi P. (2013). Striatal pre-enkephalin overexpression improves huntington’s disease symptoms in the R6/2 mouse model of huntington’s disease. *PLoS One* 8:e75099. 10.1371/journal.pone.0075099 24040390 PMC3770591

[B15] Björklund A. Dunnett S. B. (2007). Dopamine neuron systems in the brain: an update. *Trends Neurosci.* 30 194–202. 10.1016/j.tins.2007.03.006 17408759

[B16] Blandini F. (2001). The role of the subthalamic nucleus in the pathophysiology of Parkinson’s disease. *Funct. Neurol.* 16 99–106. 11996537

[B17] Bolam J. P. Pissadaki E. K. (2012). Living on the edge with too many mouths to feed: Why dopamine neurons die. *Mov. Disord.* 27 1478–1483. 10.1002/mds.25135 23008164 PMC3504389

[B18] Booth H. D. E. Hirst W. D. Wade-Martins R. (2017). The role of astrocyte dysfunction in Parkinson’s disease pathogenesis. *Trends Neurosci.* 40 358–370. 10.1016/j.tins.2017.04.001 28527591 PMC5462417

[B19] Borghammer P. (2018). How does parkinson’s disease begin? Perspectives on neuroanatomical pathways, prions, and histology. *Mov. Disord.* 33 48–57. 10.1002/mds.27138 28843014

[B20] Boumezbeur F. Mason G. F. de Graaf R. A. Behar K. L. Cline G. W. Shulman G. I. (2010). Altered brain mitochondrial metabolism in healthy aging as assessed by in vivo magnetic resonance spectroscopy. *J. Cereb. Blood Flow Metab.* 30 211–221. 10.1038/jcbfm.2009.197 19794401 PMC2949111

[B21] Bouteloup C. Desport J. C. Clavelou P. Guy N. Derumeaux-Burel H. Ferrier A. (2009). Hypermetabolism in ALS patients: an early and persistent phenomenon. *J. Neurol.* 256 1236–1242. 10.1007/s00415-009-5100-z 19306035

[B22] Braak H. Ghebremedhin E. Rüb U. Bratzke H. Del Tredici K. (2004). Stages in the development of Parkinson’s disease-related pathology. *Cell Tissue Res.* 318 121–134. 10.1007/s00441-004-0956-9 15338272

[B23] Brahmachari S. Lee S. Kim S. Yuan C. Karuppagounder S. S. Ge P. (2019). Parkin interacting substrate zinc finger protein 746 is a pathological mediator in Parkinson’s disease. *Brain* 142 2380–2401. 10.1093/brain/awz172 31237944 PMC6658849

[B24] Brandstater M. E. Lambert E. H. (1973). “Motor unit anatomy. Type and spatial arrangement of muscle fibers,” in *New Developments in Electromyography and Clinical Neurophysiology*, ed. Desmedt J. E. (Basel: S. Karger AG), 14–22. 10.1159/000394021

[B25] Brichta L. Greengard P. (2014). Molecular determinants of selective dopaminergic vulnerability in Parkinson’s disease: an update. *Front. Neuroanat.* 8:152 10.3389/fnana.2014.00152 PMC4266033 25565977

[B26] Brundin P. Ma J. Kordower J. H. (2016). How strong is the evidence that Parkinson’s disease is a prion disorder? *Curr. Opin. Neurol.* 29 459–466. 10.1097/WCO.0000000000000349 27257944 PMC5054685

[B27] Brundin P. Melki R. (2017). Prying into the prion hypothesis for Parkinson’s disease. *J. Neurosci.* 37 9808–9818. 10.1523/JNEUROSCI.1788-16.2017 29021298 PMC5637113

[B28] Brustovetsky N. Brustovetsky T. Purl K. J. Capano M. Crompton M. Dubinsky J. M. (2003). Increased susceptibility of striatal mitochondria to calcium-induced permeability transition. *J. Neurosci.* 23 4858–4867. 10.1523/JNEUROSCI.23-12-04858.2003 12832508 PMC6741171

[B29] Büeler H. (2009). Impaired mitochondrial dynamics and function in the pathogenesis of Parkinson’s disease. *Exp. Neurol.* 218 235–246. 10.1016/j.expneurol.2009.03.006 19303005

[B30] Burbulla L. F. Song P. Mazzulli J. R. Zampese E. Wong Y. C. Jeon S. (2017). Dopamine oxidation mediates mitochondrial and lysosomal dysfunction in Parkinson’s disease. *Science* 357 1255–1261. 10.1126/science.aam9080 28882997 PMC6021018

[B31] Camilleri A. Zarb C. Caruana M. Ostermeier U. Ghio S. Högen T. (2013). Mitochondrial membrane permeabilisation by amyloid aggregates and protection by polyphenols. *Biochim. Biophys. Acta Biomembr.* 1828 2532–2543. 10.1016/j.bbamem.2013.06.026 23817009

[B32] Carriedo S. G. Sensi S. L. Yin H. Z. Weiss J. H. (2000). AMPA exposures induce mitochondrial Ca 2+ overload and ROS generation in spinal motor neurons in vitro. *J. Neurosci.* 20 240–250. 10.1523/JNEUROSCI.20-01-00240.2000 10627601 PMC6774118

[B33] Castelli V. Benedetti E. Antonosante A. Catanesi M. Pitari G. Ippoliti R. (2019). Neuronal cells rearrangement during aging and neurodegenerative disease: metabolism, oxidative stress and organelles dynamic. *Front. Mol. Neurosci.* 12:132 10.3389/fnmol.2019.00132 PMC6546816 31191244

[B34] Castilho R. F. Hansson O. Ward M. W. Budd S. L. Nicholls D. G. (1998). Mitochondrial control of acute glutamate excitotoxicity in cultured cerebellar granule cells. *J. Neurosci.* 18 10277–10286. 10.1523/JNEUROSCI.18-24-10277.1998 9852565 PMC6793348

[B35] Chauhan A. Jeans A. F. (2015). Is parkinson’s disease truly a prion-like disorder? An appraisal of current evidence. *Neurol. Res. Int.* 2015 10.1155/2015/345285 25653875 PMC4310229

[B36] Chen X. Pan W. (2015). The treatment strategies for neurodegenerative diseases by integrative medicine. *Integr. Med. Int.* 1 223–225. 10.1159/000381546

[B37] Chip S. Nitsch C. Wellmann S. Kapfhammer J. P. (2013). Subfield-specific neurovascular remodeling in the entorhino-hippocampal- organotypic slice culture as a response to oxygen-glucose deprivation and excitotoxic cell death. *J. Cereb. Blood Flow Metab.* 33 508–518. 10.1038/jcbfm.2012.190 23232944 PMC3618384

[B38] Chung C. Y. Seo H. Sonntag K. C. Brooks A. Lin L. Isacson O. (2005). Cell type-specific gene expression of midbrain dopaminergic neurons reveals molecules involved in their vulnerability and protection. *Hum. Mol. Genet.* 14 1709–1725. 10.1093/hmg/ddi178 15888489 PMC2674782

[B39] Clarke L. E. Liddelow S. A. Chakraborty C. Münch A. E. Heiman M. Barres B. A. (2018). Normal aging induces A1-like astrocyte reactivity. *Proc. Natl. Acad. Sci. U.S.A.* 115 E1896–E1905. 10.1073/pnas.1800165115 29437957 PMC5828643

[B40] Connolly N. M. C. Düssmann H. Anilkumar U. Huber H. J. Prehn J. H. M. (2014). Single-cell imaging of bioenergetic responses to neuronal excitotoxicity and oxygen and glucose deprivation. *J. Neurosci.* 34 10192–10205. 10.1523/JNEUROSCI.3127-13.2014 25080581 PMC6608276

[B41] Conradi S. Ronnevi L. O. (1993). Selective vulnerability of alpha motor neurons in ALS: relation to autoantibodies toward acetylcholinesterase (AChE) in ALS patients. *Brain Res. Bull.* 30 369–371. 10.1016/0361-9230(93)90267-f 8457885

[B42] Craft S. (2007). Insulin resistance and Alzheimer’s disease pathogenesis: potential mechanisms and implications for treatment. *Curr. Alzheimer Res.* 4 147–152. 10.2174/156720507780362137 17430239

[B43] Craft S. (2009). The role of metabolic disorders in alzheimer disease and vascular dementia. *Arch. Neurol.* 66 300–305. 10.1001/archneurol.2009.27 19273747 PMC2717716

[B44] Craft S. Peskind E. Schwartz M. W. Schellenberg G. D. Raskind M. Porte D. (1998). Cerebrospinal fluid and plasma insulin levels in Alzheimer’s disease: relationship to severity of dementia and apolipoprotein E genotype. *Neurology* 50 164–168. 10.1212/WNL.50.1.164 9443474

[B45] Cudkowicz M. Kowall N. W. (1990). Degeneration of pyramidal projection neurons in Huntington’s disease cortex. *Ann. Neurol.* 27 200–204. 10.1002/ana.410270217 2138444

[B46] Dai W. Lopez O. L. Carmichael O. T. Becker J. T. Kuller L. H. Gach H. M. (2009). Mild cognitive impairment and alzheimer disease: patterns of altered cerebral blood flow at MR imaging. *Radiology* 250 856–866. 10.1148/radiol.2503080751 19164119 PMC2680168

[B47] Damier P. Hirsch E. C. Agid Y. Graybiel A. M. (1999). The substantia nigra of the human brain. *Brain* 122 1437–1448. 10.1093/brain/122.8.1437 10430830

[B48] Dauer W. Przedborski S. (2003). Parkinson’s disease: mechanisms and models. *Neuron* 39 889–909. 10.1016/S0896-6273(03)00568-3 12971891

[B49] De Jong G. I Farkas E. Stienstra C. M. Plass J. R. M. Keijser J. N. de la Torre J. C. (1999). Cerebral hypoperfusion yields capillary damage in the hippocampal CA1 area that correlates with spatial memory impairment. *Neuroscience* 91 203–210. 10.1016/S0306-4522(98)00659-9 10336071

[B50] De Jong G. I. Naber P. A. Van Der Zee E. A. Thompson L. T. Disterhoft J. F. Luiten P. G. M. (1996). Age-related loss of calcium binding proteins in rabbit hippocampus. *Neurobiol. Aging* 17 459–465. 10.1016/0197-4580(96)00030-9 8725908

[B51] de Leon M. J. Ferris S. H. George A. E. Reisberg B. Christman D. R. Kricheff I. I. (1983). Computed tomography and positron emission transaxial tomography evaluations of normal aging and Alzheimer’s disease. *J. Cereb. Blood Flow Metab.* 3 391–394. 10.1038/jcbfm.1983.57 6603463

[B52] De Vos K. J. Chapman A. L. Tennant M. E. Manser C. Tudor E. L. Lau K.-F. (2007). Familial amyotrophic lateral sclerosis-linked SOD1 mutants perturb fast axonal transport to reduce axonal mitochondria content. *Hum. Mol. Genet.* 16 2720–2728. 10.1093/hmg/ddm226 17725983 PMC4516806

[B53] Deister C. A. Teagarden M. A. Wilson C. J. Paladini C. A. (2009). An intrinsic neuronal oscillator underlies dopaminergic neuron bursting. *J. Neurosci.* 29 15888–15897. 10.1523/JNEUROSCI.4053-09.2009 20016105 PMC2824818

[B54] Derejko M. Sławek J. Lass P. Nyka W. M. (2001). Cerebral blood flow changes in Parkinson?s disease associated with dementia. *Nucl. Med. Rev. Cent. East. Eur.* 4 123–127. 14600899

[B55] Devi L. Raghavendran V. Prabhu B. M. Avadhani N. G. Anandatheerthavarada H. K. (2008). Mitochondrial import and accumulation of α-synuclein impair complex I in human dopaminergic neuronal cultures and Parkinson disease brain. *J. Biol. Chem.* 283 9089–9100. 10.1074/jbc.M710012200 18245082 PMC2431021

[B56] Devine M. J. Kittler J. T. (2018). Mitochondria at the neuronal presynapse in health and disease. *Nat. Rev. Neurosci.* 19 63–80. 10.1038/nrn.2017.170 29348666

[B57] Dharshini S. A. P. Taguchi Y. Gromiha M. M. (2019). Investigating the energy crisis in Alzheimer disease using transcriptome study. *Sci. Rep.* 9:18509. 10.1038/s41598-019-54782-y 31811163 PMC6898285

[B58] Di Maio R. Barrett P. J. Hoffman E. K. Barrett C. W. Zharikov A. Borah A. (2016). α-synuclein binds to TOM20 and inhibits mitochondrial protein import in Parkinson’s disease. *Sci. Transl. Med.* 8:342ra78. 10.1126/scitranslmed.aaf3634 27280685 PMC5016095

[B59] Dupuis L. Pradat P.-F. Ludolph A. C. Loeffler J.-P. (2011). Energy metabolism in amyotrophic lateral sclerosis. *Lancet Neurol.* 10 75–82. 10.1016/S1474-4422(10)70224-6 21035400

[B60] Durães F. Pinto M. Sousa E. (2018). Old drugs as new treatments for neurodegenerative diseases. *Pharmaceuticals* 11:44. 10.3390/ph11020044 29751602 PMC6027455

[B61] Duvernoy H. M. Cattin F. Risold P. Y. Vannson J. L. Gaudron M. (2013). *The human Hippocampus: Functional Anatomy, Vascularization and Serial Sections With MRI, Fourth Edition.* Berlin: Springer 10.1007/978-3-642-33603-4

[B62] Ehrlich M. E. (2012). Huntington’s disease and the striatal medium spiny neuron: cell-autonomous and non-cell-autonomous mechanisms of disease. *Neurotherapeutics* 9 270–284. 10.1007/s13311-012-0112-2 22441874 PMC3337013

[B63] Esposito Z. Belli L. Toniolo S. Sancesario G. Bianconi C. Martorana A. (2013). Amyloid β, glutamate, excitotoxicity in alzheimer’s disease: are we on the right track? *CNS Neurosci. Ther.* 19 549–555. 10.1111/cns.12095 23593992 PMC6493397

[B64] Estakhr J. Abazari D. Frisby K. McIntosh J. M. Nashmi R. (2017). Differential control of dopaminergic excitability and locomotion by cholinergic inputs in mouse substantia nigra. *Curr. Biol.* 27 1900.e4–1914.e4. 10.1016/j.cub.2017.05.084 28648825 PMC5529215

[B65] Estrada Sánchez A. M. Mejía-Toiber J. Massieu L. (2008). Excitotoxic neuronal death and the pathogenesis of huntington’s disease. *Arch. Med. Res.* 39 265–276. 10.1016/j.arcmed.2007.11.011 18279698

[B66] Fearnley J. M. Lees A. J. (1991). Ageing and Parkinson’s disease: substantia nigra regional selectivity. *Brain* 114(Pt 5) 2283–2301. 10.1093/brain/114.5.2283 1933245

[B67] Federico A. Cardaioli E. Da Pozzo P. Formichi P. Gallus G. N. Radi E. (2012). Mitochondria, oxidative stress and neurodegeneration. *J. Neurol. Sci.* 322 254–262. 10.1016/j.jns.2012.05.030 22669122

[B68] Foehring R. C. Zhang X. F. Lee J. C. F. Callaway J. C. (2009). Endogenous calcium buffering capacity of substantia nigral dopamine neurons. *J. Neurophysiol.* 102 2326–2333. 10.1152/jn.00038.2009 19675297 PMC2775382

[B69] Förstl H. Kurz A. (1999). Clinical features of Alzheimer’s disease. *Eur. Arch. Psychiatry Clin. Neurosci.* 249 288–290. 10.1007/s004060050101 10653284

[B70] Freeman L. R. Keller J. N. (2012). Oxidative stress and cerebral endothelial cells: Regulation of the blood-brain-barrier and antioxidant based interventions. *Biochim. Biophys. Acta Mol. Basis Dis.* 1822 822–829. 10.1016/j.bbadis.2011.12.009 22206999 PMC3412391

[B71] Fu H. Hardy J. Duff K. E. (2018). Selective vulnerability in neurodegenerative diseases. *Nat. Neurosci.* 21 1350–1358. 10.1038/s41593-018-0221-2 30250262 PMC6360529

[B72] Fuller S. Steele M. Münch G. (2010). Activated astroglia during chronic inflammation in Alzheimer’s disease-Do they neglect their neurosupportive roles? *Mutat. Res. Fundam. Mol. Mech. Mutagen.* 690 40–49. 10.1016/j.mrfmmm.2009.08.016 19748514

[B73] Gabuzda D. Busciglio J. Chen L. B. Matsudaira P. Yankner B. A. (1994). Inhibition of energy metabolism alters the processing of amyloid precursor protein and induces a potentially amyloidogenic derivative. *J. Biol. Chem.* 269 13623–13628. 8175797

[B74] Gan L. Cookson M. R. Petrucelli L. La Spada A. R. (2018). Converging pathways in neurodegeneration, from genetics to mechanisms. *Nat. Neurosci.* 21 1300–1309. 10.1038/s41593-018-0237-7 30258237 PMC6278826

[B75] Garbuzova-Davis S. Sanberg P. R. (2014). Blood-CNS barrier impairment in ALS patients versus an animal model. *Front. Cell. Neurosci.* 8:21. 10.3389/fncel.2014.00021 24550780 PMC3910123

[B76] Gardiner P. F. (1993). Physiological properties of motoneurons innervating different muscle unit types in rat gastrocnemius. *J. Neurophysiol.* 69 1160–1170. 10.1152/jn.1993.69.4.1160 8492155

[B77] Gaspar P. Ben Jelloun N. Febvret A. (1994). Sparing of the dopaminergic neurons containing Calbindin-D28k and of the dopaminergic mesocortical projections in weaver mutant mice. *Neuroscience* 61 293–305. 10.1016/0306-4522(94)90232-1 7969910

[B78] Gasparini L. Racchi M. Benussi L. Curti D. Binetti G. Bianchetti A. (1997). Effect of energy shortage and oxidative stress on amyloid precursor protein metabolism in COS cells. *Neurosci. Lett.* 231 113–117. 10.1016/S0304-3940(97)00536-3 9291153

[B79] Genç B. Jara J. H. Schultz M. C. Manuel M. Stanford M. J. Gautam M. (2016). Absence of UCHL 1 function leads to selective motor neuropathy. *Ann. Clin. Transl. Neurol.* 3 331–345. 10.1002/acn3.298 27231703 PMC4863746

[B80] Gerfen C. R. Baimbridge K. G. Miller J. J. (1985). The neostriatal mosaic: compartmental distribution of calcium-binding protein and parvalbumin in the basal ganglia of the rat and monkey. *Proc. Natl. Acad. Sci. U.S.A.* 82 8780–8784. 10.1073/pnas.82.24.8780 3909155 PMC391521

[B81] German D. C. Manaye K. Smith W. K. Woodward D. J. Saper C. B. (1989). Midbrain dopaminergic cell loss in Parkinson’s disease: computer visualization. *Ann. Neurol.* 26 507–514. 10.1002/ana.410260403 2817827

[B82] German D. C. Manaye K. F. Sonsalla P. K. Brooks B. A. (1992). Midbrain dopaminergic cell loss in Parkinson’s disease and MPTP-induced parkinsonism: sparing of calbindin-D28k-containing cells. *Ann. N. Y. Acad. Sci.* 648 42–62. 10.1111/j.1749-6632.1992.tb24523.x 1353337

[B83] Giguère N. Delignat-Lavaud B. Herborg F. Voisin A. Li Y. Jacquemet V. (2019). Increased vulnerability of nigral dopamine neurons after expansion of their axonal arborization size through D2 dopamine receptor conditional knockout. *PLoS Genet.* 15:e1008352. 10.1371/journal.pgen.1008352 31449520 PMC6730950

[B84] Glass C. K. Saijo K. Winner B. Marchetto M. C. Gage F. H. (2010). Mechanisms underlying inflammation in neurodegeneration. *Cell* 140 918–934. 10.1016/j.cell.2010.02.016 20303880 PMC2873093

[B85] Glass M. Dragunow M. Faull R. L. M. (2000). The pattern of neurodegeneration in Huntington’s disease: a comparative study of cannabinoid, dopamine, adenosine and GABAA receptor alterations in the human basal ganglia in Huntington’s disease. *Neuroscience* 97 505–519. 10.1016/S0306-4522(00)00008-7 10828533

[B86] Goldin A. Beckman J. A. Schmidt A. M. Creager M. A. (2006). Advanced glycation end products: sparking the development of diabetic vascular injury. *Circulation* 114 597–605. 10.1161/CIRCULATIONAHA.106.621854 16894049

[B87] Goldman S. M. (2014). Environmental toxins and Parkinson’s disease. *Annu. Rev. Pharmacol. Toxicol.* 54 141–164. 10.1146/annurev-pharmtox-011613-135937 24050700

[B88] Grafton S. T. Mazziotta J. C. Pahl J. J. George-Hyslop P. St Haines J. L. Gusella J. (1992). Serial changes of cerebral glucose metabolism and caudate size in persons at risk for huntington’s disease. *Arch. Neurol.* 49 1161–1167. 10.1001/archneur.1992.00530350075022 1444883

[B89] Greene J. G. Dingledine R. Greenamyre J. T. (2005). Gene expression profiling of rat midbrain dopamine neurons: implications for selective vulnerability in parkinsonism. *Neurobiol. Dis.* 18 19–31. 10.1016/j.nbd.2004.10.003 15649693

[B90] Gregory J. M. Livesey M. R. McDade K. Selvaraj B. T. Barton S. K. Chandran S. (2019). Dysregulation of AMPA receptor subunit expression in sporadic ALS post−mortem brain. *J. Pathol.* 250 67–78. 10.1002/path.5351 path.5351. 31579943 PMC6973025

[B91] Gu M. Gash M. T. Mann V. M. Javoy-Agid F. Cooper J. M. Schapira A. H. V. (1996). Mitochondrial defect in Huntington’s disease caudate nucleus. *Ann. Neurol.* 39 385–389. 10.1002/ana.410390317 8602759

[B92] Guan J. Pavlovic D. Dalkie N. Waldvogel H. J. O’Carroll S. J. Green C. R. (2013). Vascular degeneration in parkinsons disease. *Brain Pathol.* 23 154–164. 10.1111/j.1750-3639.2012.00628.x 22897695 PMC8029297

[B93] Gundersen V. (2010). Protein aggregation in Parkinson’s disease. *Acta Neurol. Scand.* 122 82–87. 10.1111/j.1600-0404.2010.01382.x 20586742

[B94] Haberman R. P. Branch A. Gallagher M. (2017). Targeting neural hyperactivity as a treatment to stem progression of late-onset Alzheimer’s disease. *Neurotherapeutics* 14 662–676. 10.1007/s13311-017-0541-z 28560709 PMC5509635

[B95] Haddad D. Nakamura K. (2015). Understanding the susceptibility of dopamine neurons to mitochondrial stressors in Parkinson’s disease. *FEBS Lett.* 589 3702–3713. 10.1016/j.febslet.2015.10.021 26526613 PMC4679488

[B96] Halestrap A. P. Woodfield K. Y. Connern C. P. (1997). Oxidative stress, thiol reagents, and membrane potential modulate the mitochondrial permeability transition by affecting nucleotide binding to the adenine nucleotide translocase. *J. Biol. Chem.* 272 3346–3354. 10.1074/jbc.272.6.3346 9013575

[B97] Han I. You Y. Kordower J. H. Brady S. T. Morfini G. A. (2010). Differential vulnerability of neurons in Huntington’s disease: the role of cell type-specific features. *J. Neurochem.* 113 1073–1091. 10.1111/j.1471-4159.2010.06672.x 20236390 PMC2890032

[B98] Hansson O. Petersén Å Leist M. Nicotera P. Castilho R. F. Brundin P. (1999). Transgenic mice expressing a Huntington’s disease mutation are resistant to quinolinic acid-induced striatal excitotoxicity. *Proc. Natl. Acad. Sci. U.S.A.* 96 8727–8732. 10.1073/pnas.96.15.8727 10411943 PMC17584

[B99] Hasselbalch S. G. Oberg G. Sørensen S. A. Andersen A. R. Waldemar G. Schmidt J. F. (1992). Reduced regional cerebral blood flow in Huntington’s disease studied by SPECT. *J. Neurol. Neurosurg. Psychiatry* 55 1018–1023. 10.1136/jnnp.55.11.1018 1469396 PMC1015285

[B100] Hawkes C. H. Del Tredici K. Braak H. (2007). Parkinson’s disease: a dual-hit hypothesis. *Neuropathol. Appl. Neurobiol.* 33 599–614. 10.1111/j.1365-2990.2007.00874.x 17961138 PMC7194308

[B101] Hedreen J. C. Peyser C. E. Folstein S. E. Ross C. A. (1991). Neuronal loss in layers V and VI of cerebral cortex in Huntington’s disease. *Neurosci. Lett.* 133 257–261. 10.1016/0304-3940(91)90583-F 1840078

[B102] Hegedus J. Putman C. T. Gordon T. (2007). Time course of preferential motor unit loss in the SOD1G93A mouse model of amyotrophic lateral sclerosis. *Neurobiol. Dis.* 28 154–164. 10.1016/j.nbd.2007.07.003 17766128

[B103] Heng M. Y. Detloff P. J. Wang P. L. Tsien J. Z. Albin R. L. (2009). In vivo evidence for NMDA receptor-mediated excitotoxicity in a murine genetic model of huntington disease. *J. Neurosci.* 29 3200–3205. 10.1523/JNEUROSCI.5599-08.2009 19279257 PMC6666431

[B104] Her L. S. Goldstein L. S. B. (2008). Enhanced sensitivity of striatal neurons to axonal transport defects induced by mutant huntingtin. *J. Neurosci.* 28 13662–13672. 10.1523/JNEUROSCI.4144-08.2008 19074039 PMC6671757

[B105] Hering T. Birth N. Taanman J.-W. Orth M. (2015). Selective striatal mtDNA depletion in end-stage Huntington’s disease R6/2 mice. *Exp. Neurol.* 266 22–29. 10.1016/j.expneurol.2015.02.004 25682918

[B106] Hirsch E. Graybiel A. M. Agid Y. A. (1988). Melanized dopaminergic neurons are differentially susceptible to degeneration in Parkinson’s disease. *Nature* 334 345–348. 10.1038/334345a0 2899295

[B107] Hock C. Villringer K. Müller-Spahn F. Wenzel R. Heekeren H. Schuh-Hofer S. (1997). Decrease in parietal cerebral hemoglobin oxygenation during performance of a verbal fluency task in patients with Alzheimer’s disease monitored by means of near-infrared spectroscopy (NIRS) - Correlation with simultaneous rCBF-PET measurements. *Brain Res.* 755 293–303. 10.1016/S0006-8993(97)00122-4 9175896

[B108] Hof P. R. Morrison J. H. (2004). The aging brain: morphomolecular senescence of cortical circuits. *Trends Neurosci.* 27 607–613. 10.1016/j.tins.2004.07.013 15374672

[B109] Hof P. R. Rosenthal R. E. Fiskum G. (1996). Distribution of neurofilament protein and calcium-binding proteins parvalbumin, calbindin, and calretinin in the canine hippocampus. *J. Chem. Neuroanat.* 11 1–12. 10.1016/0891-0618(96)00117-2 8841885

[B110] Huisman M. H. B. Seelen M. Van Doormaal P. T. C. De Jong S. W. De Vries J. H. M. Van Der Kooi A. J. (2015). Effect of presymptomatic body mass index and consumption of fat and alcohol on amyotrophic lateral sclerosis. *JAMA Neurol.* 72 1155–1162. 10.1001/jamaneurol.2015.1584 26280944

[B111] Humbert S. Bryson E. A. Cordelières F. P. Connors N. C. Datta S. R. Finkbeiner S. (2002). The IGF-1/Akt pathway is neuroprotective in Huntington’s disease and involves huntingtin phosphorylation by Akt. *Dev. Cell* 2 831–837. 10.1016/S1534-5807(02)00188-0 12062094

[B112] Hunot S. Vila M. Teismann P. Davis R. J. Hirsch E. C. Przedborski S. (2004). JNK-mediated induction of cyclooxygenase 2 is required for neurodegeneration in a mouse model of Parkinson’s disease. *Proc. Natl. Acad. Sci. U.S.A.* 101 665–670. 10.1073/pnas.0307453101 14704277 PMC327205

[B113] Ilieva H. Polymenidou M. Cleveland D. W. (2009). Non–cell autonomous toxicity in neurodegenerative disorders: ALS and beyond. *J. Cell Biol.* 187 761–772. 10.1083/jcb.200908164 19951898 PMC2806318

[B114] Ishiguro H. Yamada K. Sawada H. Nishii K. Ichino N. Sawada M. (2001). Age-dependent and tissue-specific CAG repeat instability occurs in mouse knock-in for a mutant Huntington’s disease gene. *J. Neurosci. Res.* 65 289–297. 10.1002/jnr.1153 11494364

[B115] Iwangoff P. Armbruster R. Enz A. Meier-Ruge W. (1980). Glycolytic enzymes from human autoptic brain cortex: normal aged and demented cases. *Mech. Ageing Dev.* 14 203–209. 10.1016/0047-6374(80)90120-7 6259457

[B116] Jackson T. C. Rani A. Kumar A. Foster T. C. (2009). Regional hippocampal differences in AKT survival signaling across the lifespan: Implications for CA1 vulnerability with aging. *Cell Death Differ.* 16 439–448. 10.1038/cdd.2008.171 19039330 PMC2680608

[B117] Jiang R. Diaz-Castro B. Looger L. L. Khakh B. S. (2016). Dysfunctional calcium and glutamate signaling in striatal astrocytes from Huntington’s disease model mice. *J. Neurosci.* 36 3453–3470. 10.1523/JNEUROSCI.3693-15.2016 27013675 PMC4804005

[B118] Johri A. Beal M. F. (2012). Mitochondrial dysfunction in neurodegenerative diseases. *J. Pharmacol. Exp. Ther.* 342 619–630. 10.1124/jpet.112.192138 22700435 PMC3422529

[B119] Kalaria R. N. Harik S. I. (1989). Reduced glucose transporter at the blood-brain barrier and in cerebral cortex in Alzheimer disease. *J. Neurochem.* 53 1083–1088. 10.1111/j.1471-4159.1989.tb07399.x 2769254

[B120] Kamphuis W. Middeldorp J. Kooijman L. Sluijs J. A. Kooi E. J. Moeton M. (2014). Glial fibrillary acidic protein isoform expression in plaque related astrogliosis in Alzheimer’s disease. *Neurobiol. Aging* 35 492–510. 10.1016/j.neurobiolaging.2013.09.035 24269023

[B121] Kanak D. J. Rose G. M. Zaveri H. P. Patrylo P. R. (2013). Altered network timing in the CA3-CA1 circuit of hippocampal slices from aged mice. *PLoS One* 8:e61364. 10.1371/journal.pone.0061364 23593474 PMC3620228

[B122] Kaplan A. Spiller K. J. Towne C. Kanning K. C. Choe G. T. Geber A. (2014). Neuronal matrix metalloproteinase-9 is a determinant of selective neurodegeneration. *Neuron* 81 333–348. 10.1016/j.neuron.2013.12.009 24462097 PMC6015650

[B123] Kawahara Y. Kwak S. Sun H. Ito K. Hashida H. Aizawa H. (2003). Human spinal motoneurons express low relative abundance of GluR2 mRNA: an implication for excitotoxicity in ALS. *J. Neurochem.* 85 680–689. 10.1046/j.1471-4159.2003.01703.x 12694394

[B124] Kawamura Y. Dyck P. J. Shimono M. Okazaki H. Tateishi J. Doi H. (1981). Morphometric comparison of the vulnerability of peripheral motor and sensory neurons in amyotrophic lateral sclerosis. *J. Neuropathol. Exp. Neurol.* 40 667–675. 10.1097/00005072-198111000-00008 7299423

[B125] Kayser E. B. Sedensky M. M. Morgan P. G. (2016). Region-specific defects of respiratory capacities in the Ndufs4(KO) mouse brain. *PLoS One* 11:e0148219. 10.1371/journal.pone.0148219 26824698 PMC4732614

[B126] Keller A. F. Gravel M. Kriz J. (2009). Live imaging of amyotrophic lateral sclerosis pathogenesis: disease onset is characterized by marked induction of GFAP in schwann cells. *Glia* 57 1130–1142. 10.1002/glia.20836 19115383

[B127] Kennedy L. (2000). Dramatic mutation instability in HD mouse striatum: does polyglutamine load contribute to cell-specific vulnerability in Huntington’s disease? *Hum. Mol. Genet.* 9 2539–2544. 10.1093/hmg/9.17.2539 11030759

[B128] Kernell D. Zwaagstra B. (1981). Input conductance, axonal conduction velocity and cell size among hindlimb motoneurones of the cat. *Brain Res.* 204 311–326. 10.1016/0006-8993(81)90591-6 7459634

[B129] Kew J. J. Leigh P. N. Playford E. D. Passingham R. E. Goldstein L. H. Frackowiak R. S. (1993). Cortical function in amyotrophic lateral sclerosis. *Brain* 116 655–680. 10.1093/brain/116.3.655 8513396

[B130] Khakh B. S. (2019). Astrocyte–neuron interactions in the striatum: insights on identity, form, and function. *Trends Neurosci.* 42 617–630. 10.1016/j.tins.2019.06.003 31351745 PMC6741427

[B131] Kiaei M. (2013). New hopes and challenges for treatment of neurodegenerative disorders: great opportunities for young neuroscientists. *Basic Clin. Neurosci.* 4 3–4 25337322 PMC4202552

[B132] Kish S. J. Bergeron C. Rajput A. Dozic S. Mastrogiacomo F. Chang L.-J., et al. (1992). Brain cytochrome oxidase in alzheimer’s Disease. *J. Neurochem.* 59 776–779. 10.1111/j.1471-4159.1992.tb09439.x 1321237

[B133] Koprich J. B. Reske-Nielsen C. Mithal P. Isacson O. (2008). Neuroinflammation mediated by IL-1beta increases susceptibility of dopamine neurons to degeneration in an animal model of Parkinson’s disease. *J. Neuroinflammation* 5:8. 10.1186/1742-2094-5-8 18304357 PMC2292163

[B134] Kordower J. H. Chu Y. Hauser R. A. Freeman T. B. Olanow C. W. (2008). Lewy body-like pathology in long-term embryonic nigral transplants in Parkinson’s disease. *Nat. Med.* 14 504–506. 10.1038/nm1747 18391962

[B135] Korovila I. Hugo M. Castro J. P. Weber D. Höhn A. Grune T. (2017). Proteostasis, oxidative stress and aging. *Redox Biol.* 13 550–567. 10.1016/j.redox.2017.07.008 28763764 PMC5536880

[B136] Kure S. Tominaga T. Yoshimoto T. Tada K. Narisawa K. (1991). Glutamate triggers internucleosomal DNA cleavage in neuronal cells. *Biochem. Biophys. Res. Commun.* 179 39–45. 10.1016/0006-291X(91)91330-F 1679329

[B137] Kushner P. D. Stephenson D. T. Wright S. (1991). Reactive astrogliosis is widespread in the subcortical white matter of amyotrophic lateral sclerosis brain. *J. Neuropathol. Exp. Neurol.* 50 263–277. 10.1097/00005072-199105000-00008 2022968

[B138] Kusindarta D. L. Wihadmadyatami H. Haryanto A. (2018). The analysis of hippocampus neuronal density (CA1 and CA3) after Ocimum sanctum ethanolic extract treatment on the young adulthood and middle age rat model. *Vet. World* 11 135–140. 10.14202/vetworld.2018.135-140 29657393 PMC5891864

[B139] Kuwert T. Boecker H. Titz H. Herzog H. Wang B. C. Nayak U. (1993). Striatal glucose consumption in chorea-free subjects at risk of Huntington’s disease. *J. Neurol.* 241 31–36. 10.1007/BF00870669 8138819

[B140] Lalić N. M. Marić J. Svetel M. Jotić A. Stefanova E. Lalić K. (2008). Glucose homeostasis in Huntington disease: abnormalities in insulin sensitivity and early-phase insulin secretion. *Arch. Neurol.* 65 476–480. 10.1001/archneur.65.4.476 18413469

[B141] Lee J. M. Pinto R. M. Gillis T. St. Claire J. C. Wheeler V. C. (2011). Quantification of age-dependent somatic CAG repeat instability in Hdh CAG knock-in mice reveals different expansion dynamics in striatum and liver. *PLoS One* 6:e23647. 10.1371/journal.pone.0023647 21897851 PMC3163641

[B142] Lee Y. Chakraborty S. Meininger C. J. Muthuchamy M. (2018). Insulin resistance disrupts cell integrity, mitochondrial function, and inflammatory signaling in lymphatic endothelium. *Microcirculation* 25:e12492. 10.1111/micc.12492 30025187 PMC6170722

[B143] Lee Y. Morrison B. M. Li Y. Lengacher S. Farah M. H. Hoffman P. N. (2012). Oligodendroglia metabolically support axons and contribute to neurodegeneration. *Nature* 487 443–448. 10.1038/nature11314 22801498 PMC3408792

[B144] Lee Y. J. Yan B. C. Park J. H. Ahn J. H. Kim I. H. Lee J.-C. (2013). Differences of calcium binding proteins immunoreactivities in the young hippocampal CA1 region from the adult following transient ischemic damage. *J. Neurol. Sci.* 326 40–47. 10.1016/j.jns.2012.12.026 23357314

[B145] Li J. Y. Englund E. Holton J. L. Soulet D. Hagell P. Lees A. J. (2008). Lewy bodies in grafted neurons in subjects with Parkinson’s disease suggest host-to-graft disease propagation. *Nat. Med.* 14 501–503. 10.1038/nm1746 18391963

[B146] Liang C. L. Nelson O. Yazdani U. Pasbakhsh P. German D. C. (2004). Inverse relationship between the contents of neuromelanin pigment and the vesicular monoamine transporter-2: human midbrain dopamine neurons. *J. Comp. Neurol.* 473 97–106. 10.1002/cne.20098 15067721

[B147] Liang C. L. Sinton C. M. German D. C. (1996). Midbrain dopaminergic neurons in the mouse: co-localization with calbindin-D(28K) and calretinin. *Neuroscience* 75 523–533. 10.1016/0306-4522(96)00228-X 8931015

[B148] Liddelow S. A. Guttenplan K. A. Clarke L. E. Bennett F. C. Bohlen C. J. Schirmer L. (2017). Neurotoxic reactive astrocytes are induced by activated microglia. *Nature* 541 481–487. 10.1038/nature21029 28099414 PMC5404890

[B149] Liu F. C. Graybiel A. M. (1992). Transient calbindin-D28k-positive systems in the telencephalon: ganglionic eminence, developing striatum and cerebral cortex. *J. Neurosci.* 12 674–690. 10.1523/jneurosci.12-02-00674.1992 1740695 PMC6575618

[B150] Liu X. Kim C. N. Yang J. Jemmerson R. Wang X. (1996). Induction of apoptotic program in cell-free extracts: requirement for dATP and cytochromec. *Cell* 86 147–157. 10.1016/S0092-8674(00)80085-9 8689682

[B151] Liu Z. Lv C. Zhao W. Song Y. Pei D. Xu T. (2012). NR2B-containing NMDA receptors expression and their relationship to apoptosis in hippocampus of Alzheimer’s disease-like rats. *Neurochem. Res.* 37 1420–1427. 10.1007/s11064-012-0726-0 22359056

[B152] Luthi-Carter R. Strand A. Peters N. L. Solano S. M. Hollingsworth Z. R. Menon A. S. (2000). Decreased expression of striatal signaling genes in a mouse model of Huntington’s disease. *Hum. Mol. Genet.* 9 1259–1271. 10.1093/hmg/9.9.1259 10814708

[B153] Marambaud P. Dreses-Werringloer U. Vingtdeux V. (2009). Calcium signaling in neurodegeneration. *Mol. Neurodegener.* 4:20. 10.1186/1750-1326-4-20 19419557 PMC2689218

[B154] Marques A. Dutheil F. Durand E. Rieu I. Mulliez A. Fantini M. L. (2018). Glucose dysregulation in Parkinson’s disease: too much glucose or not enough insulin? *Parkinsonism Relat. Disord.* 55 122–127. 10.1016/j.parkreldis.2018.05.026 29866628

[B155] Matheoud D. Cannon T. Voisin A. Penttinen A.-M. Ramet L. Fahmy A. M. (2019). Intestinal infection triggers Parkinson’s disease-like symptoms in Pink1-/- mice. *Nature* 571 565–569. 10.1038/s41586-019-1405-y 31316206

[B156] Mattiasson G. Friberg H. Hansson M. Elmér E. Wieloch T. (2003). Flow cytometric analysis of mitochondria from CA1 and CA3 regions of rat hippocampus reveals differences in permeability transition pore activation. *J. Neurochem.* 87 532–544. 10.1046/j.1471-4159.2003.02026.x 14511130

[B157] Mattiazzi M. D’Aurelio M. Gajewski C. D. Martushova K. Kiaei M. Flint Beal M. (2002). Mutated human SOD1 causes dysfunction of oxidative phosphorylation in mitochondria of transgenic mice. *J. Biol. Chem.* 277 29626–29633. 10.1074/jbc.M203065200 12050154

[B158] Mattson M. P. Magnus T. (2006). Ageing and neuronal vulnerability. *Nat. Rev. Neurosci.* 7 278–294. 10.1038/nrn1886 16552414 PMC3710114

[B159] Maxwell N. Castro R. W. Sutherland N. M. Vaughan K. L. Szarowicz M. D. de Cabo R. (2018). α-Motor neurons are spared from aging while their synaptic inputs degenerate in monkeys and mice. *Aging Cell* 17:12726. 10.1111/acel.12726 29397579 PMC5847869

[B160] McGeer P. L. McGeer E. G. (2008). Glial reactions in Parkinson’s disease. *Mov. Disord.* 23 474–483. 10.1002/mds.21751 18044695

[B161] Medina L. Figueredo-Cardenas G. Reiner A. (1996a). Differential abundance of superoxide dismutase in interneurons versus projection neurons and in matrix versus striosome neurons in monkey striatum. *Brain Res.* 708 59–70. 10.1016/0006-8993(95)01320-2 8720860

[B162] Medina L. Figueredo-Cardenas G. Rothstein J. D. Reiner A. (1996b). Differential abundance of glutamate transporter subtypes in amyotrophic lateral sclerosis (ALS)-vulnerable versus ALS-resistant brain stem motor cell groups. *Exp. Neurol.* 142 287–295. 10.1006/exnr.1996.0198 8934560

[B163] Medinas D. B. Cabral-Miranda F. Hetz C. (2019). ER stress links aging to sporadic ALS. *Aging (Albany. N. Y.)* 11 5–6. 10.18632/aging.101705 30612121 PMC6339799

[B164] Medinas D. B. Rozas P. Traub F. M. Woehlbier U. Brown R. H. Bosco D. A. (2018). Endoplasmic reticulum stress leads to accumulation of wild-type SOD1 aggregates associated with sporadic amyotrophic lateral sclerosis. *Proc. Natl. Acad. Sci. U.S.A.* 115 8209–8214. 10.1073/pnas.1801109115 30038021 PMC6094144

[B165] Mendez I. Sanchez-Pernaute R. Cooper O. Viñuela A. Ferrari D. Björklund L. (2005). Cell type analysis of functional fetal dopamine cell suspension transplants in the striatum and substantia nigra of patients with Parkinson’s disease. *Brain* 128 1498–1510. 10.1093/brain/awh510 15872020 PMC2610438

[B166] Mendez I. Vĩuela A. Astradsson A. Mukhida K. Hallett P. Robertson H. (2008). Dopamine neurons implanted into people with Parkinson’s disease survive without pathology for 14 years. *Nat. Med.* 14 507–509. 10.1038/nm1752 18391961 PMC2656682

[B167] Menzies F. M. Ince P. G. Shaw P. J. (2002). Mitochondrial involvement in amyotrophic lateral sclerosis. *Neurochem. Int.* 40 543–551. 10.1016/s0197-0186(01)00125-5 11850111

[B168] Mietelska-Porowska A. Wasik U. Goras M. Filipek A. Niewiadomska G. (2014). Tau protein modifications and interactions: their role in function and dysfunction. *Int. J. Mol. Sci.* 15 4671–4713. 10.3390/ijms15034671 24646911 PMC3975420

[B169] Miller J. A. Woltjer R. L. Goodenbour J. M. Horvath S. Geschwind D. H. (2013). Genes and pathways underlying regional and cell type changes in Alzheimer’s disease. *Genome Med.* 5:48. 10.1186/gm452 23705665 PMC3706780

[B170] Miyazaki K. Ohta Y. Nagai M. Morimoto N. Kurata T. Takehisa Y. (2011). Disruption of neurovascular unit prior to motor neuron degeneration in amyotrophic lateral sclerosis. *J. Neurosci. Res.* 89 718–728. 10.1002/jnr.22594 21337372

[B171] Mizuseki K. Royer S. Diba K. Buzsáki G. (2012). Activity dynamics and behavioral correlates of CA3 and CA1 hippocampal pyramidal neurons. *Hippocampus* 22 1659–1680. 10.1002/hipo.22002 22367959 PMC3718552

[B172] Morigaki R. Goto S. (2017). Striatal vulnerability in huntington’s disease: Neuroprotection versus neurotoxicity. *Brain Sci.* 7 2–25. 10.3390/brainsci7060063 28590448 PMC5483636

[B173] Muddapu V. R. Mandali A. Chakravarthy V. S. Ramaswamy S. (2019). A computational model of loss of dopaminergic cells in Parkinson’s disease due to glutamate-induced excitotoxicity. *Front. Neural Circuits* 13:11. 10.3389/FNCIR.2019.00011 30858799 PMC6397878

[B174] Nayak D. Roth T. L. McGavern D. B. (2014). Microglia development and function. *Annu. Rev. Immunol.* 32 367–402. 10.1146/annurev-immunol-032713-120240 24471431 PMC5001846

[B175] Nijssen J. Comley L. H. Hedlund E. (2017). Motor neuron vulnerability and resistance in amyotrophic lateral sclerosis. *Acta Neuropathol.* 133 863–885. 10.1007/s00401-017-1708-8 28409282 PMC5427160

[B176] Novelli A. Reilly J. A. Lysko P. G. Henneberry R. C. (1988). Glutamate becomes neurotoxic via the N-methyl-d-aspartate receptor when intracellular energy levels are reduced. *Brain Res.* 451 205–212. 10.1016/0006-8993(88)90765-2 2472189

[B177] O’ Neill C. (2013). PI3-kinase/Akt/mTOR signaling: impaired on/off switches in aging, cognitive decline and Alzheimer’s disease. *Exp. Gerontol.* 48 647–653. 10.1016/j.exger.2013.02.025 23470275

[B178] Oh M. M. Oliveira F. A. Waters J. Disterhoft J. F. (2013). Altered calcium metabolism in aging CA1 hippocampal pyramidal neurons. *J. Neurosci.* 33 7905–7911. 10.1523/JNEUROSCI.5457-12.2013 23637181 PMC3679661

[B179] Oh M. M. Simkin D. Disterhoft J. F. (2016). Intrinsic hippocampal excitability changes of opposite signs and different origins in CA1 and CA3 pyramidal neurons underlie aging-related cognitive deficits. *Front. Syst. Neurosci.* 10:52. 10.3389/fnsys.2016.00052 27375440 PMC4899460

[B180] Olanow C. W. Brundin P. (2013). Parkinson’s disease and alpha synuclein: is Parkinson’s disease a prion-like disorder? *Mov. Disord.* 28 31–40. 10.1002/mds.25373 23390095

[B181] Olsen L. K. Dowd E. McKernan D. P. (2018). A role for viral infections in Parkinson’s etiology? *Neuronal Signal.* 2:NS20170166 10.1042/NS20170166 PMC7373231 32714585

[B182] Ormazabal V. Nair S. Elfeky O. Aguayo C. Salomon C. Zuñiga F. A. (2018). Association between insulin resistance and the development of cardiovascular disease. *Cardiovasc. Diabetol.* 17:122. 10.1186/s12933-018-0762-4 30170598 PMC6119242

[B183] Ouyang Y.-B. Voloboueva L. A. Xu L.-J. Giffard R. G. (2007). Selective dysfunction of hippocampal CA1 astrocytes contributes to delayed neuronal damage after transient forebrain ischemia. *J. Neurosci.* 27 4253–4260. 10.1523/JNEUROSCI.0211-07.2007 17442809 PMC3140959

[B184] Pacelli C. Giguère N. Bourque M. J. Lévesque M. Slack R. S. Trudeau L. É (2015). Elevated mitochondrial bioenergetics and axonal arborization size are key contributors to the vulnerability of dopamine neurons. *Curr. Biol.* 25 2349–2360. 10.1016/j.cub.2015.07.050 26320949

[B185] Padurariu M. Ciobica A. Mavroudis I. Fotiou D. Baloyannis S. (2012a). Hippocampal neuronal loss in the CA1 and CA3 areas of Alzheimer’s disease patients. *Psychiatr. Danub.* 24 152–158. 22706413

[B186] Padurariu M. Ciobica A. Mavroudis I. Fotiou D. Baloyannis S. (2012b). Hippocampal neuronal loss in the Ca1 and Ca3 areas of Alzheimer’s disease patients. *Psychiatr Danub* 24 152–158. 22706413

[B187] Pahapill P. A. Lozano A. M. (2000). The pedunculopontine nucleus and Parkinson’s disease. *Brain* 123 1767–1783. 10.1093/brain/123.9.1767 10960043

[B188] Pathak D. Shields L. Y. Mendelsohn B. A. Haddad D. Lin W. Gerencser A. A. (2015). The role of mitochondrially derived ATP in synaptic vesicle recycling. *J. Biol. Chem.* 290 22325–22336. 10.1074/jbc.M115.656405 26126824 PMC4566209

[B189] Perry E. K. Perry R. H. Tomlinson B. E. Blessed G. Gibson P. H. (1980). Coenzyme a-acetylating enzymes in Alzheimer’s disease: possible cholinergic ‘compartment’ of pyruvate dehydrogenase. *Neurosci. Lett.* 18 105–110. 10.1016/0304-3940(80)90220-7 6133246

[B190] Perurena O. H. Festoff B. W. (1987). Reduction in insulin receptors in amyotrophic lateral sclerosis correlates with reduced insulin sensitivity. *Neurology* 37 1375–1379. 10.1212/wnl.37.8.1375 3614662

[B191] Peter D. Liu Y. Sternini C. de Giorgio R. Brecha N. Edwards R. (1995). Differential expression of two vesicular monoamine transporters. *J. Neurosci.* 15 6179–6188. 10.1523/JNEUROSCI.15-09-06179.1995 7666200 PMC6577657

[B192] Petersen C. C. H. Malenka R. C. Nicoll R. A. Hopfield J. J. (1998). All-or-none potentiation at CA3-CA1 synapses. *Proc. Natl. Acad. Sci. U.S.A.* 95 4732–4737. 10.1073/pnas.95.8.4732 9539807 PMC22559

[B193] Philips T. Bento-Abreu A. Nonneman A. Haeck W. Staats K. Geelen V. (2013). Oligodendrocyte dysfunction in the pathogenesis of amyotrophic lateral sclerosis. *Brain* 136 471–482. 10.1093/brain/aws339 23378219 PMC3572934

[B194] Philips T. Rothstein J. D. (2014). Glial cells in amyotrophic lateral sclerosis. *Exp. Neurol.* 262(Pt B) 111–120. 10.1016/j.expneurol.2014.05.015 24859452 PMC4241182

[B195] Pissadaki E. K. Bolam J. P. (2013). The energy cost of action potential propagation in dopamine neurons: clues to susceptibility in Parkinson’s disease. *Front. Comput. Neurosci.* 7:13. 10.3389/fncom.2013.00013 23515615 PMC3600574

[B196] Post M. R. Lieberman O. J. Mosharov E. V. (2018). Can interactions between α-synuclein, dopamine and calcium explain selective neurodegeneration in Parkinson’s disease? *Front. Neurosci.* 12:161 10.3389/fnins.2018.00161 PMC5861202 29593491

[B197] Potier B. Krzywkowski P. Lamour Y. Dutar P. (1994). Loss of calbindin-immunoreactivity in CA1 hippocampal stratum radiatum and stratum lacunosum-moleculare interneurons in the aged rat. *Brain Res.* 661 181–188. 10.1016/0006-8993(94)91195-9 7834368

[B198] Poulin J. F. Laforest S. Drolet G. (2014). Enkephalin downregulation in the nucleus accumbens underlies chronic stress-induced anhedonia. *Stress* 17 88–96. 10.3109/10253890.2013.850669 24090157 PMC4457518

[B199] Pun S. Santos A. F. Saxena S. Xu L. Caroni P. (2006). Selective vulnerability and pruning of phasic motoneuron axons in motoneuron disease alleviated by CNTF. *Nat. Neurosci.* 9 408–419. 10.1038/nn1653 16474388

[B200] Radenovic L. Korenic A. Maleeva G. Osadchenko I. Kovalenko T. Skibo G. (2011). Comparative ultrastructural analysis of mitochondria in the CA1 and CA3 hippocampal pyramidal cells following global ischemia in mongolian Gerbils. *Anat. Rec.* 294 1057–1065. 10.1002/ar.21390 21538930

[B201] Ravisankar P. Dhanavardhan K. Prathyusha K. Rajan K. V. (2018). Stem cell therapy role in neurodegenerative disorders. *Arch. Ment. Heal.* 19 3–8. 10.4103/amh.amh_10_18

[B202] Reeve A. K. Grady J. P. Cosgrave E. M. Bennison E. Chen C. Hepplewhite P. D. (2018). Mitochondrial dysfunction within the synapses of substantia nigra neurons in Parkinson’s disease. *npj Park. Dis* 4:9. 10.1038/s41531-018-0044-6 29872690 PMC5979968

[B203] Reiner A. Albin R. L. Anderson K. D. D’Amato C. J. Penney J. B. Young A. B. (1988). Differential loss of striatal projection neurons in Huntington disease. *Proc. Natl. Acad. Sci. U.S.A.* 85 5733–5737. 10.1073/pnas.85.15.5733 2456581 PMC281835

[B204] Reitz C. (2013). Dyslipidemia and the risk of Alzheimer’s disease. *Curr. Atheroscler. Rep.* 15:307. 10.1007/s11883-012-0307-3 23328907 PMC3564220

[B205] Restelli L. M. Oettinghaus B. Halliday M. Agca C. Licci M. Sironi L. (2018). Neuronal mitochondrial dysfunction activates the integrated stress response to induce fibroblast growth factor 21. *Cell Rep.* 24 1407–1414. 10.1016/j.celrep.2018.07.023 30089252 PMC6092266

[B206] Ribeiro M. Silva A. C. Rodrigues J. Naia L. Rego A. C. (2013). Oxidizing effects of exogenous stressors in Huntington’s disease knock-in striatal cells—protective effect of cystamine and creatine. *Toxicol. Sci.* 136 487–499. 10.1093/toxsci/kft199 24008831

[B207] Richfield E. K. Maguire−Zeiss K. A. Vonkeman H. E. Voorn P. (1995). Preferential loss of preproenkephalin versus preprotachykinin neurons from the striatum of Huntington’s disease patients. *Ann. Neurol.* 38 852–861. 10.1002/ana.410380605 8526457

[B208] Rocha S. M. Cristovão A. C. Campos F. L. Fonseca C. P. Baltazar G. (2012). Astrocyte-derived GDNF is a potent inhibitor of microglial activation. *Neurobiol. Dis.* 47 407–415. 10.1016/j.nbd.2012.04.014 22579772

[B209] Rodríguez J. J. Noristani H. N. Hilditch T. Olabarria M. Yeh C. Y. Witton J. (2013). Increased densities of resting and activated microglia in the dentate gyrus follow senile plaque formation in the CA1 subfield of the hippocampus in the triple transgenic model of Alzheimer’s disease. *Neurosci. Lett.* 552 129–134. 10.1016/j.neulet.2013.06.036 23827221

[B210] Rodriguez M. C. Obeso J. A. Olanow C. W. (1998). Subthalamic nucleus-mediated excitotoxicity in Parkinson’s disease: a target for neuroprotection. *Ann. Neurol.* 44 S175–S188. 9749591 10.1002/ana.410440726

[B211] Ross C. A. Poirier M. A. (2004). Protein aggregation and neurodegenerative disease. *Nat. Med.* 10 S10–S17. 10.1038/nm1066 15272267

[B212] Rothman S. M. Olney J. W. (1986). Glutamate and the pathophysiology of hypoxic-ischemic brain damage. *Ann. Neurol.* 19 105–111. 10.1002/ana.410190202 2421636

[B213] Rudow G. O’Brien R. Savonenko A. V. Resnick S. M. Zonderman A. B. Pletnikova O. (2008). Morphometry of the human substantia nigra in ageing and Parkinson’s disease. *Acta Neuropathol.* 115 461–470. 10.1007/s00401-008-0352-8 18297291 PMC2431149

[B214] Ruegsegger C. Maharjan N. Goswami A. Filézac, de L’Etang A. Weis J. (2016). Aberrant association of misfolded SOD1 with Na+/K+ATPase-α3 impairs its activity and contributes to motor neuron vulnerability in ALS. *Acta Neuropathol.* 131 427–451. 10.1007/s00401-015-1510-4 26619836

[B215] Saxena S. Cabuy E. Caroni P. (2009). A role for motoneuron subtype-selective ER stress in disease manifestations of FALS mice. *Nat. Neurosci.* 12 627–636. 10.1038/nn.2297 19330001

[B216] Schapira A. H. (2008). Mitochondria in the aetiology and pathogenesis of Parkinson’s disease. *Lancet Neurol.* 7 97–109. 10.1016/S1474-4422(07)70327-7 18093566

[B217] Shin J. H. Ko H. S. Kang H. Lee Y. Lee Y. II. (2011). PARIS (ZNF746) repression of PGC-1α contributes to neurodegeneration in parkinson’s disease. *Cell* 144 689–702. 10.1016/j.cell.2011.02.010 21376232 PMC3063894

[B218] Shin J.-Y. Fang Z.-H. Yu Z.-X. Wang C.-E. Li S.-H. Li X.-J. (2005). Expression of mutant huntingtin in glial cells contributes to neuronal excitotoxicity. *J. Cell Biol.* 171 1001–1012. 10.1083/jcb.200508072 16365166 PMC2171327

[B219] Simkin D. Hattori S. Ybarra N. Musial T. F. Buss E. W. Richter H. (2015). Aging-related hyperexcitability in CA3 pyramidal neurons is mediated by enhanced A-Type K+ channel function and expression. *J. Neurosci.* 35 13206–13218. 10.1523/JNEUROSCI.0193-15.2015 26400949 PMC4579378

[B220] Smith I. D. Grace A. A. (1992). Role of the subthalamic nucleus in the regulation of nigral dopamine neuron activity. *Synapse* 12 287–303. 10.1002/syn.890120406 1465742

[B221] Smith Y. Charara A. Parent A. (1996). Synaptic innervation of midbrain dopaminergic neurons by glutamate-enrriched terminals in the squirrel monkey. *J. Comp. Neurol.* 364 231–253. 10.1002/(SICI)1096-9861(19960108)364:2<231::AID-CNE4<3.0.CO;2-6 8788247

[B222] Song J. Kim J. (2016). Degeneration of dopaminergic neurons due to metabolic alterations and Parkinson’s disease. *Front. Aging Neurosci.* 8:65 10.3389/fnagi.2016.00065 PMC4811934 27065205

[B223] Sorbi S. Bird E. D. Blass J. P. (1983). Decreased pyruvate dehydrogenase complex activity in Huntington and Alzheimer brain. *Ann. Neurol.* 13 72–78. 10.1002/ana.410130116 6219611

[B224] Sorolla M. A. Reverter-Branchat G. Tamarit J. Ferrer I. Ros J. Cabiscol E. (2008). Proteomic and oxidative stress analysis in human brain samples of Huntington disease. *Free Radic. Biol. Med.* 45 667–678. 10.1016/j.freeradbiomed.2008.05.014 18588971

[B225] Stanika R. I. Winters C. A. Pivovarova N. B. Andrews S. B. (2010). Differential NMDA receptor-dependent calcium loading and mitochondrial dysfunction in CA1 vs. CA3 hippocampal neurons. *Neurobiol. Dis.* 37 403–411. 10.1016/j.nbd.2009.10.020 19879359 PMC2818520

[B226] Stifani N. (2014). Motor neurons and the generation of spinal motor neuron diversity. *Front. Cell. Neurosci* 8:293. 10.3389/fncel.2014.00293 25346659 PMC4191298

[B227] Su B. Wang X. Zheng L. Perry G. Smith M. A. Zhu X. (2010). Abnormal mitochondrial dynamics and neurodegenerative diseases. *Biochim. Biophys. Acta Mol. Basis Dis.* 1802 135–142. 10.1016/j.bbadis.2009.09.013 19799998 PMC2790543

[B228] Surmeier D. J. Guzman J. N. Sanchez-Padilla J. Goldberg J. A. (2010). What causes the death of dopaminergic neurons in Parkinson’s disease? *Prog. Brain Res.* 183 59–77. 10.1016/S0079-6123(10)83004-3 20696315

[B229] Surmeier D. J. Obeso J. A. Halliday G. M. (2017). Parkinson’s Disease Is Not Simply a Prion Disorder. *J. Neurosci.* 37 9799–9807. 10.1523/JNEUROSCI.1787-16.2017 29021297 PMC5637112

[B230] Sweeney M. D. Kisler K. Montagne A. Toga A. W. Zlokovic B. V. (2018a). The role of brain vasculature in neurodegenerative disorders. *Nat. Neurosci.* 21 1318–1331. 10.1038/s41593-018-0234-x 30250261 PMC6198802

[B231] Sweeney M. D. Sagare A. P. Zlokovic B. V. (2018b). Blood–brain barrier breakdown in Alzheimer disease and other neurodegenerative disorders. *Nat. Rev. Neurol.* 14 133–150. 10.1038/nrneurol.2017.188 29377008 PMC5829048

[B232] Swerdlow R. H. Parks J. K. Cassarino D. S. Trimmer P. A. Miller S. W. Maguire D. J. (1998). Mitochondria in sporadic amyotrophic lateral sclerosis. *Exp. Neurol.* 153 135–142. 10.1006/exnr.1998.6866 9743575

[B233] Tan S. Wood M. Maher P. (2002). Oxidative stress induces a form of programmed cell death with characteristics of both apoptosis and necrosis in neuronal cells. *J. Neurochem.* 71 95–105. 10.1046/j.1471-4159.1998.71010095.x 9648855

[B234] Tansey M. G. Goldberg M. S. (2010). Neuroinflammation in Parkinson’s disease: Its role in neuronal death and implications for therapeutic intervention. *Neurobiol. Dis.* 37 510–518. 10.1016/J.NBD.2009.11.004 19913097 PMC2823829

[B235] Teismann P. Tieu K. Choi D.-K. Wu D.-C. Naini A. Hunot S. (2003). Cyclooxygenase-2 is instrumental in Parkinson’s disease neurodegeneration. *Proc. Natl. Acad. Sci. U.S.A.* 100 5473–5478. 10.1073/pnas.0837397100 12702778 PMC154369

[B236] Timmons S. Coakley M. F. Moloney A. M. O’ Neill C. (2009). Akt signal transduction dysfunction in Parkinson’s disease. *Neurosci. Lett.* 467 30–35. 10.1016/J.NEULET.2009.09.055 19800394

[B237] Tomé S. Dandelot E. (2017). “Genetic modifiers of CAG.CTG repeat instability in Huntington’s disease mouse models,” in *Huntington’s Disease - Molecular Pathogenesis and Current Models*, ed. Tunali N. E. (London: InTech Open). 10.5772/66438

[B238] Umegaki H. Roth G. S. Ingram D. K. (2008). Aging of the striatum: mechanisms and interventions. *Age (Dordr.)* 30 251–261. 10.1007/s11357-008-9066-z 19424849 PMC2585651

[B239] Vaarmann A. Kovac S. Holmström K. M. Gandhi S. Abramov A. Y. (2013). Dopamine protects neurons against glutamate-induced excitotoxicity. *Cell Death Dis* 4:e455. 10.1038/cddis.2012.194 23303129 PMC3563982

[B240] Van Den Bosch L. Vandenberghe W. Klaassen H. Van Houtte E. Robberecht W. (2000). Ca2+-permeable AMPA receptors and selective vulnerability of motor neurons. *J. Neurol. Sci.* 180 29–34. 10.1016/S0022-510X(00)00414-7 11090861

[B241] Van Laar V. S. Berman S. B. (2009). Mitochondrial dynamics in Parkinson’s disease. *Exp. Neurol.* 218 247–256. 10.1016/j.expneurol.2009.03.019 19332061 PMC2752687

[B242] Vandoorne T. De Bock K. Van Den Bosch L. (2018). Energy metabolism in ALS: an underappreciated opportunity? *Acta Neuropathol.* 135 489–509. 10.1007/s00401-018-1835-x 29549424 PMC5978930

[B243] Vielhaber S. (2000). Mitochondrial DNA abnormalities in skeletal muscle of patients with sporadic amyotrophic lateral sclerosis. *Brain* 123 1339–1348. 10.1093/brain/123.7.1339 10869047

[B244] Waak J. Weber S. S. Waldenmaier A. Görner K. Alunni-Fabbroni M. Schell H. (2009). Regulation of astrocyte inflammatory responses by the Parkinson’s disease-associated gene DJ-1. *FASEB J.* 23 2478–2489. 10.1096/fj.08-125153 19276172

[B245] Wainger B. J. Kiskinis E. Mellin C. Wiskow O. Han S. S. W. Sandoe J. (2014). Intrinsic membrane hyperexcitability of amyotrophic lateral sclerosis patient-derived motor neurons. *Cell Rep.* 7 1–11. 10.1016/j.celrep.2014.03.019 24703839 PMC4023477

[B246] Wang R. Reddy P. H. (2017). Role of Glutamate and NMDA Receptors in Alzheimer’s Disease. *J. Alzheimers Dis.* 57 1041–1048. 10.3233/JAD-160763 27662322 PMC5791143

[B247] Wang X. Pal R. Chen X. Limpeanchob N. Kumar K. N. Michaelis E. K. (2005). High intrinsic oxidative stress may underlie selective vulnerability of the hippocampal CA1 region. *Mol. Brain Res.* 140 120–126. 10.1016/j.molbrainres.2005.07.018 16137784

[B248] Wang X. Yu S. Gao S.-J. Hu J.-P. Wang Y. Liu H.-X. (2014). Insulin inhibits Abeta production through modulation of APP processing in a cellular model of Alzheimer’s disease. *Neuro Endocrinol. Lett.* 35 224–229. 24977973

[B249] Wilde G. J. Pringle A. K. Wright P. Iannotti F. (1997). Differential vulnerability of the CA1 and CA3 subfields of the hippocampus to superoxide and hydroxyl radicals in vitro. *J. Neurochem.* 69 883–886. 10.1046/j.1471-4159.1997.69020883.x 9231752

[B250] Willette A. A. Bendlin B. B. Starks E. J. Birdsill A. C. Johnson S. C. Christian B. T. (2015). Association of Insulin Resistance With Cerebral Glucose Uptake in Late Middle-Aged Adults at Risk for Alzheimer Disease. *JAMA Neurol.* 72 1013–1020. 10.1001/jamaneurol.2015.0613 26214150 PMC4570876

[B251] Winklhofer K. F. Haass C. (2010). Mitochondrial dysfunction in Parkinson’s disease. *Biochim. Biophys. Acta* 1802 29–44. 10.1016/j.bbadis.2009.08.013 19733240

[B252] Zambon F. Cherubini M. Fernandes H. J. R. Lang C. Ryan B. J. Volpato V. (2019). Cellular α-synuclein pathology is associated with bioenergetic dysfunction in Parkinson’s iPSC-derived dopamine neurons. *Hum. Mol. Genet.* 28 2001–2013. 10.1093/hmg/ddz038 30753527 PMC6548224

[B253] Zecca L. Wilms H. Geick S. Claasen J.-H. Brandenburg L.-O. Holzknecht C. (2008). Human neuromelanin induces neuroinflammation and neurodegeneration in the rat substantia nigra: implications for Parkinson’s disease. *Acta Neuropathol.* 116 47–55. 10.1007/s00401-008-0361-7 18343932

[B254] Zhang F. Ström A.-L. Fukada K. Lee S. Hayward L. J. Zhu H. (2007). Interaction between familial amyotrophic lateral sclerosis (ALS)-linked SOD1 mutants and the dynein complex. *J. Biol. Chem.* 282 16691–16699. 10.1074/jbc.M609743200 17403682

[B255] Zhang W. Phillips K. Wielgus A. R. Liu J. Albertini A. Zucca F. A. (2011). Neuromelanin activates microglia and induces degeneration of dopaminergic neurons: implications for progression of Parkinson’s disease. *Neurotox. Res.* 19 63–72. 10.1007/s12640-009-9140-z 19957214 PMC3603276

[B256] Zhao W. Varghese M. Yemul S. Pan Y. Cheng A. Marano P. (2011). Peroxisome proliferator activator receptor gamma coactivator-1alpha (PGC-1α) improves motor performance and survival in a mouse model of amyotrophic lateral sclerosis. *Mol. Neurodegener.* 6:51. 10.1186/1750-1326-6-51 21771318 PMC3156746

[B257] Zhou J. Gennatas E. D. Kramer J. H. Miller B. L. Seeley W. W. (2012). Predicting regional neurodegeneration from the healthy brain functional connectome. *Neuron* 73 1216–1227. 10.1016/j.neuron.2012.03.004 22445348 PMC3361461

[B258] Zhu X. Smith M. A. Honda K. Aliev G. Moreira P. I. Nunomura A. (2007). Vascular oxidative stress in Alzheimer disease. *J. Neurol. Sci.* 257 240–246. 10.1016/j.jns.2007.01.039 17337008 PMC1952687

[B259] Zweifel L. S. Parker J. G. Lobb C. J. Rainwater A. Wall V. Fadok J. P. (2009). Disruption of NMDAR-dependent burst firing by dopamine neurons provides selective assessment of phasic dopamine-dependent behavior. *Proc. Natl. Acad. Sci. U.S.A.* 106 7281–7288. 10.1073/pnas.0813415106 19342487 PMC2678650

